# Adult Mortality Attributable to Preventable Risk Factors for Non-Communicable Diseases and Injuries in Japan: A Comparative Risk Assessment

**DOI:** 10.1371/journal.pmed.1001160

**Published:** 2012-01-24

**Authors:** Nayu Ikeda, Manami Inoue, Hiroyasu Iso, Shunya Ikeda, Toshihiko Satoh, Mitsuhiko Noda, Tetsuya Mizoue, Hironori Imano, Eiko Saito, Kota Katanoda, Tomotaka Sobue, Shoichiro Tsugane, Mohsen Naghavi, Majid Ezzati, Kenji Shibuya

**Affiliations:** 1 Department of Global Health Policy, Graduate School of Medicine, University of Tokyo, Tokyo, Japan; 2 Epidemiology and Prevention Division, Research Center for Cancer Prevention and Screening, National Cancer Center, Tokyo, Japan; 3 Department of Social and Environmental Medicine, Osaka University Graduate School of Medicine, Osaka, Japan; 4 International University of Health and Welfare Graduate School, Tokyo, Japan; 5 Kitasato Clinical Research Center, Kitasato University School of Medicine, Sagamihara, Japan; 6 Department of Diabetes and Metabolic Medicine, National Center for Global Health and Medicine, Tokyo, Japan; 7 Department of Epidemiology and International Health, National Center for Global Health and Medicine, Tokyo, Japan; 8 Cancer Information Services and Surveillance Division, Center for Cancer Control and Information Services, National Cancer Center, Tokyo, Japan; 9 Institute for Health Metrics and Evaluation, University of Washington, Seattle, Washington, United States of America; 10 MRC-HPA Centre for Environment and Health, Department of Epidemiology and Biostatistics, School of Public Health, Imperial College London, London, United Kingdom; Umeå Centre for Global Health Research, Sweden

## Abstract

**Background:**

The population of Japan has achieved the longest life expectancy in the world. To further improve population health, consistent and comparative evidence on mortality attributable to preventable risk factors is necessary for setting priorities for health policies and programs. Although several past studies have quantified the impact of individual risk factors in Japan, to our knowledge no study has assessed and compared the effects of multiple modifiable risk factors for non-communicable diseases and injuries using a standard framework. We estimated the effects of 16 risk factors on cause-specific deaths and life expectancy in Japan.

**Methods and Findings:**

We obtained data on risk factor exposures from the National Health and Nutrition Survey and epidemiological studies, data on the number of cause-specific deaths from vital records adjusted for ill-defined codes, and data on relative risks from epidemiological studies and meta-analyses. We applied a comparative risk assessment framework to estimate effects of excess risks on deaths and life expectancy at age 40 y. In 2007, tobacco smoking and high blood pressure accounted for 129,000 deaths (95% CI: 115,000–154,000) and 104,000 deaths (95% CI: 86,000–119,000), respectively, followed by physical inactivity (52,000 deaths, 95% CI: 47,000–58,000), high blood glucose (34,000 deaths, 95% CI: 26,000–43,000), high dietary salt intake (34,000 deaths, 95% CI: 27,000–39,000), and alcohol use (31,000 deaths, 95% CI: 28,000–35,000). In recent decades, cancer mortality attributable to tobacco smoking has increased in the elderly, while stroke mortality attributable to high blood pressure has declined. Life expectancy at age 40 y in 2007 would have been extended by 1.4 y for both sexes (men, 95% CI: 1.3–1.6; women, 95% CI: 1.2–1.7) if exposures to multiple cardiovascular risk factors had been reduced to their optimal levels as determined by a theoretical-minimum-risk exposure distribution.

**Conclusions:**

Tobacco smoking and high blood pressure are the two major risk factors for adult mortality from non-communicable diseases and injuries in Japan. There is a large potential population health gain if multiple risk factors are jointly controlled.

*Please see later in the article for the Editors' Summary*

## Abstract 2

Using a combination of published data and modeling, Nayu Ikeda and colleagues identify tobacco smoking and high blood pressure as major risk factors for death from noncommunicable diseases among adults in Japan.

## Editors' Summary

**Background:**

Worldwide, a small number of modifiable risk factors are responsible for many premature or preventable deaths. For example, having high blood pressure (hypertension) increases a person's risk of developing life-threatening heart problems and stroke (cardiovascular disease). Similarly, having a high blood sugar level increases the risk of developing diabetes, a chronic (long-term) disease that can lead to cardiovascular problems and kidney failure, and half of all long-term tobacco smokers in Western populations will die prematurely from diseases related to smoking, such as lung cancer. Importantly, the five major risk factors for death globally—high blood pressure, tobacco use, high blood sugar, physical inactivity, and overweight and obesity—are all modifiable. That is, lifestyle changes and dietary changes such as exercising more, reducing salt intake, and increasing fruit and vegetable intake can reduce an individual's exposure to these risk factors and one's chances of premature death. Moreover, public health programs designed to reduce a population's exposure to modifiable risk factors should reduce preventable deaths in that population.

**Why Was This Study Done?:**

In 2000, the Japanese government initiated Health Japan 21, a ten-year national health promotion campaign designed to prevent premature death from non-communicable (noninfectious) diseases and injuries. This campaign set 59 goals to monitor and improve risk factor management in the Japanese population, which has one of the longest life expectancies at birth in the world (the life expectancy of a person born in Japan in 2009 was 83.1 years). Because the campaign's final evaluation revealed deterioration or no improvement on some of these goals, the Japanese government recently released new guidelines that stress the importance of simultaneously controlling multiple risk factors for chronic diseases. However, although several studies have quantified the impacts on life expectancy and cause-specific death of individual modifiable risk factors in Japan, the effects of multiple risk factors have not been assessed. In this study, the researchers use a “comparative risk assessment” framework to estimate the effects of 16 risk factors on cause-specific deaths and life expectancy in Japan. Comparative risk assessment estimates the number of deaths that would be prevented if current distributions of risk factor exposures were changed to hypothetical optimal distributions.

**What Did the Researchers Do and Find?:**

The researchers obtained data on exposure to the selected risk factors from the 2007 Japanese National Health and Nutrition Survey and from epidemiological studies, and information on the number of deaths in 2007 from different diseases from official records. They used published studies to estimate how much each factor increases the risk of death from each disease and then used a mathematical formula to estimate the effects of the risk factors on the number of deaths in Japan and on life expectancy at age 40. In 2007, tobacco smoking and high blood pressure accounted for 129,000 and 104,000 deaths, respectively, in Japan. Physical inactivity accounted for 52,000 deaths, high blood glucose and high dietary salt intake accounted for 34,000 deaths each, and alcohol use for 31,000 deaths. Life expectancy at age 40 in 2007 would have been extended by 1.4 years for both sexes, the researchers estimate, if exposure to multiple cardiovascular risk factors had been reduced to calculated optimal distributions, or by 0.7 years if these risk factors had been reduced to the distributions defined by national guidelines and goals.

**What Do These Findings Mean?:**

These findings identify tobacco smoking and high blood pressure as the major risk factors for death from non-communicable diseases among adults in Japan, a result consistent with previous findings from the US. They also indicate that simultaneous control of multiple risk factors has great potential for producing health gains among the Japanese population. Although the researchers focused on estimating the effect of these risk factors on mortality and did not include illness and disability in this study, these findings nevertheless identify two areas of public health policy that need to be strengthened to improve health, reduce death rates, and increase life expectancy among the Japanese population. First, they highlight the need to reduce tobacco smoking, particularly among men. Second and most importantly, these findings emphasize the need to improve ongoing programs designed to help people manage multiple cardiovascular risk factors, including high blood pressure.

**Additional Information:**

Please access these websites via the online version of this summary at http://dx.doi.org/10.1371/journal.pmed.1001160.

- The US Centers for Disease Control and Prevention provides information on all aspects of healthy living
- The *World Health Report 2002—Reducing Risks, Promoting Healthy Life* provides a global analysis of how healthy life expectancy could be increased
- The American Heart Association and the American Cancer Society provide information on many important risk factors for noncommunicable diseases and include some personal stories about keeping healthy
- Details about Health Japan 21 are provided by the Japanese Ministry of Health, Labour and Welfare. Further details about this campaign are available from the World Health Organization
- MedlinePlus provides links to further resources on healthy living and on healthy aging (in English and Spanish)

## Introduction

Controlling risk factors for non-communicable diseases and external causes is essential for the improvement of adult health. Chronic diseases and injuries are the leading causes of global mortality, accounting for 63% and 9%, respectively, of 57 million deaths in 2008 [1]. The five major risk factors for deaths in the world are high blood pressure, tobacco use, high blood glucose, physical inactivity, and overweight and obesity, which contribute to non-communicable diseases and are modifiable with effective interventions [2]. In such an environment, informed decision-making on priority setting for health policies and programs needs consistent and comparative evidence about how many deaths would be averted by changing profiles of preventable risk factors in a population.

The population of Japan has the longest life expectancy at birth in the world. Life expectancy at birth for Japanese women was 54.0 y in 1947 and rapidly increased until 1986, at which point, at 81.0 y, it became the longest in the world for the first time; female life expectancy at birth also reached its highest ever worldwide figure, 86.4 y, in Japan in 2009 [3]. The continuous extension of longevity was largely explained by a decline in the rate of mortality for communicable diseases among children and young adults during the 1950s and the early 1960s and for stroke since the late 1960s [4]. Current leading causes of death are malignant neoplasm, heart disease, and cerebrovascular disease, accounting for more than 50% of total deaths in 2009 [5]. Accidental injuries and suicide have also ranked in the top ten causes of death for the past 50 y [5], and particularly suicide in the working population is a serious social problem reflecting the prolonged economic recession since the 1990s [4]. To further enhance the health status of the Japanese population, it is therefore crucial to prevent deaths from these major causes.

With the aim of increasing the nation's health through the prevention of premature deaths from lifestyle-related diseases, the Japanese government initiated a 10-y national health promotion campaign called Health Japan 21 in 2000 [6]. In this campaign, 59 indicators were established to monitor and improve the management of risk factors and diseases such as diet, smoking, and diabetes. However, the performance of Health Japan 21 was not necessarily satisfactory: there was progress on 60% of the 59 indicators, including decreasing daily salt intake, while deterioration or no improvement was observed for the remaining 40%, for example, the prevalence of overweight and obesity decreased in women aged 40–60 y but increased in men aged 20–60 y [7]. Success of national health promotion campaigns may partly depend on whether the stewardship of central and local governments exists for coordinating diverse activities and investing resources in priority areas with reference to scientific evidence on the disease burden attributable to modifiable risk factors. Although a number of past studies have quantified population-attributable fractions or impacts on life expectancy for individual risk factors in Japan [8]–[16], no study to our knowledge has used a single comprehensive framework to assess and compare these impacts across multiple risk factors.

In the present study, we therefore aimed to provide the most comprehensive and comparative assessment of preventable risk factors for mortality from non-communicable diseases and injuries in the Japanese adult population. We employed a comparative risk assessment strategy to quantify contributions of health risks to disease outcomes [17],[18]. This standard systematic approach has already been applied to examine the burden of disease and injury across major risk factors in a few other countries [19]–[22]. Using national data sources on risk exposures and cause-specific mortality, as well as epidemiologic evidence on their causal association from large-scale prospective studies and meta-analyses in Japan, this analysis identifies the most important risk factors for deaths and life expectancy at the population level; the results could inform policymakers of which risk factors need to be prioritized in formulating and revising health policies and programs.

## Methods

We estimated the number of deaths that would have been saved in 2007 if multiple risk factors had been controlled at their optimal levels as determined by a theoretical-minimum-risk exposure distribution. To quantify and compare the mortality attributable to excess health risks, we used comparative risk assessment methods that have been described in detail elsewhere [18],[20]. To summarize, we first calculated the population-attributable fraction of cause-specific mortality for each risk factor, which measures a proportional reduction in mortality that would be achieved if risk factor exposures of a population shifted to an alternative counterfactual distribution that is more favorable. We used the following formula to calculate population-attributable fractions for continuous exposure variables:
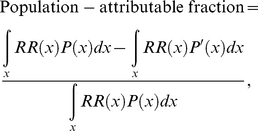
(1)where *P*(*x*) and *P*′(*x*) are actual and counterfactual distributions of exposure in the population, respectively, and *RR*(*x*) is the relative risk of mortality at exposure level *x*. The first and second terms in the numerator of this equation represent the total risk of mortality weighted by exposures in the population under current and counterfactual distributions, respectively. This approach allowed us to compute effects of all nonoptimal exposures of individuals for all risk factors in a consistent and comparable way [21]. For risks measured in multiple categories, we used the following generalized formula to calculate population-attributable fractions:
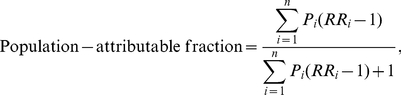
(2)where *i* signifies the level of individual categories (*i* = 1,…, *n*).

We then multiplied the number of cause-specific deaths by population-attributable fractions to estimate mortality from diseases (causes of death) associated with each risk factor. The number of deaths attributable to a single risk factor was summed across different causes to obtain the total number of deaths attributable to that risk factor. The number of deaths from a single cause, however, could not be added across risk factors, because they may be causally related and we did not account for such relationships in the estimation of population-attributable fractions of individual risk factors.

We conducted all analyses separately by sex, using Stata version 11 (StataCorp). We restricted analyses to individuals aged 30 y and over, because the number of deaths from non-communicable diseases is small for younger ages. However, we included those aged 20 to 29 y when estimating deaths from external causes attributable to alcohol use, because the burden was assumed to be substantial in this age group.

### Mortality Data

We obtained data on the number of cause-specific deaths in 2007 from vital records [23]. We applied algorithms developed for the Global Burden of Disease 2010 Study to redistribute ill-defined codes (e.g., cardiac arrest, heart failure, and senility) on death certificates that were not supposed to be underlying causes of death [24],[25]. This method enabled us to obtain valid, reliable, and comparable data on cause-specific mortality by ensuring consistency and resolving changes across revisions of the *International Statistical Classification of Diseases and Related Health Problems*.

### Selection of Risk Factors and Diseases

We included 16 risk factors in this analysis (Table 1). In the selection of risk factors paired with their relevant diseases or injuries, we employed the criteria of a previous study: (i) an availability of evidence on causality or association from high-quality epidemiological studies, (ii) an existence of interventions to modify exposures, and (iii) an availability of data on risk exposures from nationally representative surveys or large population studies [20]. We also included infection by several agents—hepatitis B virus, hepatitis C virus, the bacterium *Helicobacter pylori*, human papillomavirus, and human T-lymphotropic virus type 1 (HTLV-1)—because they are important risk factors for cancer deaths in Japan [26],[27].

**Table 1:** Risk factors and disease outcomes included in the study.

Risk Factor	Disease Outcomes
High blood glucose	IHD, stroke, diabetes mellitus
High LDL cholesterol	IHD, ischemic stroke
High blood pressure	IHD, stroke, hypertensive diseases, other cardiovascular diseases^a^
Overweight/obesity	IHD; ischemic stroke; hypertensive disease; postmenopausal breast, colon, corpus uteri, kidney, and pancreatic cancers; diabetes mellitus
Alcohol use	IHD; ischemic stroke; hemorrhagic stroke; hypertensive diseases; cardiac arrhythmias; cancers of breast, colorectal, esophagus, mouth, liver, larynx, pharynx, and selected other sites^b^; diabetes mellitus; liver cirrhosis; acute and chronic pancreatitis; road traffic injuries; falls; homicide and suicide; other injuries
Tobacco smoking	IHD; stroke; aortic aneurysms and dissection; diabetes mellitus; lung, esophagus, mouth, pharynx, stomach, liver, pancreas, cervix, bladder, kidney, and other urinary cancers; leukemia; chronic obstructive pulmonary disease; lower respiratory tract infections; asthma; tuberculosis
Physical inactivity	IHD, ischemic stroke, breast and colon cancers, diabetes mellitus
High dietary trans fatty acids	IHD
Low dietary polyunsaturated fatty acids	IHD
High dietary salt	IHD, stroke, hypertensive disease, other cardiovascular diseases^a^, stomach cancer
Low intake of fruit and vegetables	IHD; ischemic stroke; colorectal, esophagus, lung, mouth, pharynx, and stomach cancers
Hepatitis B virus	Liver cancer
Hepatitis C virus	Liver cancer
*H. pylori*	Stomach cancer
Human papillomavirus	Cervix uteri cancer
HTLV-1	Adult T-cell lymphoma/leukemia

a This category includes rheumatic heart disease, endocarditis, cardiomyopathy, aortic aneurysms, peripheral vascular disorders, and other ill-defined cardiovascular diseases.

b This category includes *International Statistical Classification of Diseases and Related Health Problems, 10th edition* (ICD-10) codes D00–D24 (except D09.9), D26–D37 (except D37.9), and D38–D48 (except D38.6, D39.9, D40.9, D41.9, and D48.9).

IHD, ischemic heart disease.

### Measures and Data Sources of Risk Factor Exposures

Table 2 lists measurements and data sources for the risk factor exposures used in this analysis, and Table 3 shows their basic statistics by sex and age group in 2007. With the exception of tobacco smoking, infections, and alcohol use related to deaths from traffic road accidents, we used individual records from the National Health and Nutrition Survey (NHNS) in 2007. NHNS was a survey based on a nationally representative probabilistic sample to provide data on the health and nutritional status of the Japanese population. This survey included an in-person interview on medication use and lifestyle-related risk factors, a physical examination by health care professionals, and self-administered questionnaires on diet and lifestyle [28].

**Table 2:** Measurements, data sources, and alternative distributions of risk exposures.

Risk Factor, Exposure Metric, Data Source^a^	Optimal	Guidelines/National Goals
**High blood glucose**		
Fasting plasma glucose (mmol/l)	4.9 (0.3)	5.6 (0.3) [66]
**High LDL cholesterol**		
LDL cholesterol (mmol/l)	2.0 (0.4)	3.1 (0.7) [54]
**High blood pressure**		
Systolic blood pressure (mm Hg)	115 (6)	130 (7) [53]
**Overweight/obesity**		
Body mass index (kg/m^2^)	21 (1)	22 (1) [67]
**Alcohol use**		
Current alcohol consumption volumes and patterns	No alcohol use^b^	
Alcohol-related road traffic accidents, national road accident data, 2004 [40]	No alcohol use	
**Tobacco smoking**		
Smoking impact ratio, vital statistics 2007 data [23]–[25], pooled cohort studies [15],[35],[36]	No smoking	
**Physical inactivity**		
Intensity of physical activity	Highly active	
**High dietary trans fatty acids**		
Percent of total calories from dietary trans fatty acids	0.5 (0.05)	
**Low dietary polyunsaturated fatty acids**		
Percent of total calories from dietary polyunsaturated fatty acids	10 (1)	
**High dietary salt**		
Dietary sodium adjusted for total calories (g/d)	0.5 (0.05)	10 (1) [7]
**Low intake of fruit and vegetables**		
Dietary fruit and vegetable intake adjusted for total calories (g/d)	600 (50)	350 (29) [7]
**Hepatitis B virus**		
Seropositivity for hepatitis B surface antigen, blood donors' cohort, 1991–1993 [37]	No infection	
**Hepatitis C virus**		
Seropositivity for antibody to hepatitis C, blood donors' cohort, 1991–1993 [37]	No infection	
***H. pylori***		
Seropositivity for anti–*H. pylori* immunoglobulin G, multi-center study, late 1990s [38]	No infection	

Values are means, with standard deviations in parentheses.

a We obtained exposure data from the 2007 National Health and Nutrition Survey [28] unless stated otherwise.

b The optimal category for liver cancer and suicide was “occasional drinkers” because previous studies used it as the reference category for estimation of relative risks.

**Table 3:** Exposure to risk factors by sex and age group in 2007.

Sex, Risk Factor	Age														
	30–44 y			45–59 y			60–69 y			70–79 y			≥80 y		
	*n* a	Mean	SE	*n* a	Mean	SE	*n* a	Mean	SE	*n* a	Mean	SE	*n* a	Mean	SE
**Men**															
Fasting plasma glucose (mmol/l)	300	5.4	0.1	374	5.7	0.0	411	6.0	0.1	339	5.9	0.0	107	5.9	0.1
LDL cholesterol (mmol/l)	300	3.3	0.0	375	3.4	0.0	413	3.1	0.0	340	3.0	0.0	108	2.9	0.1
Systolic blood pressure (mm Hg)	312	124.2	0.8	394	133.9	0.9	427	140.9	0.9	359	142.2	1.0	116	144.1	1.8
Body mass index (kg/m^2^)	673	23.9	0.1	777	23.8	0.1	620	23.8	0.1	470	23.6	0.2	155	22.6	0.3
Dietary TFA (% of total calories)	806	0.3	0.0	858	0.3	0.0	664	0.2	0.0	517	0.2	0.0	179	0.3	0.0
Dietary PUFA (% of total calories)	806	5.7	0.1	858	5.6	0.1	664	5.3	0.1	517	5.1	0.1	179	5.0	0.1
Dietary SFA (% of total calories)	806	6.8	0.1	858	6.2	0.1	664	5.7	0.1	517	5.6	0.1	179	5.9	0.2
Dietary salt intake (g/d)	806	11.4	0.2	858	12.3	0.2	664	12.6	0.2	517	12.2	0.2	179	10.9	0.3
Fruit and vegetable intake (g/d)	804	288.6	6.0	856	342.5	6.6	663	432.3	8.8	515	446.8	9.6	178	463.5	15.9
Never or former drinkers (%)^b^	850	26.6	1.5	950	25.9	1.4	699	28.9	1.7	525	40.8	2.1	184	55.4	3.7
Alcohol-related accidents/four-wheel vehicle road traffic accidents, 2004 (%) [40] c		1.7			1.7			1.7			1.7			1.7	
Have intense physical activity (%)	808	34.8	1.7	869	34.5	1.6	667	28.8	1.8	518	42.1	2.2	179	21.8	3.1
Never or former smokers (%)	850	45.2	1.7	946	53.6	1.6	698	65.9	1.8	524	78.6	1.8	184	82.1	2.8
Smoking impact ratio		0.0			0.6			0.5			0.5			0.7	
Hepatitis B virus (%) [37]		0.9			0.9			0.9			0.6			0.6	
Hepatitis C virus (%) [37]		0.6			1.6			2.6			7.9			7.9	
*H. pylori* (%) [38]		23.6			47.4			66.1			73.4			72.6	
**Women**															
Fasting plasma glucose (mmol/l)	563	5.3	0.0	620	5.7	0.0	523	5.9	0.0	408	5.9	0.0	154	5.9	0.1
LDL cholesterol (mmol/l)	565	2.9	0.0	622	3.4	0.0	523	3.5	0.0	410	3.3	0.0	154	3.2	0.1
Systolic blood pressure (mm Hg)	527	112.4	0.6	652	128.1	0.8	560	135.8	0.8	433	138.9	0.8	170	143.2	1.4
Body mass index (kg/m^2^)	874	21.4	0.1	905	22.7	0.1	723	23.3	0.1	534	23.1	0.2	248	22.4	0.3
Dietary TFA (% of total calories)	955	0.4	0.0	957	0.3	0.0	762	0.3	0.0	561	0.3	0.0	285	0.2	0.0
Dietary PUFA (% of total calories)	955	5.9	0.1	957	6.0	0.1	762	5.6	0.1	561	5.3	0.1	285	5.3	0.1
Dietary SFA (% of total calories)	955	7.8	0.1	957	7.0	0.1	762	6.2	0.1	561	5.9	0.1	285	5.7	0.2
Dietary salt intake (g/d)	955	9.6	0.1	957	10.7	0.1	762	10.9	0.2	561	10.6	0.2	285	10.0	0.2
Fruit and vegetable intake (g/d)	951	346.0	6.1	957	460.2	7.6	761	541.3	9.0	561	522.8	9.7	284	490.3	13.2
Never or former drinkers (%)^b^	1,014	54.9	1.6	1,047	61.1	1.5	795	75.5	1.5	579	83.4	1.5	310	88.7	1.8
Alcohol-related accidents/four-wheel vehicle road traffic accidents, 2004 (%) [40] c		1.7			1.7			1.7			1.7			1.7	
Have intense physical activity (%)	958	36.3	1.6	959	40.9	1.6	765	39.0	1.8	562	45.4	2.1	286	21.3	2.4
Never or former smokers (%)	1,014	81.8	1.2	1,047	87.1	1.0	794	92.1	1.0	579	96.5	0.8	310	95.5	1.2
Smoking impact ratio		0.0			0.1			0.2			0.2			0.2	
Hepatitis B virus (%) [37]		0.5			0.5			0.5			0.6			0.6	
Hepatitis C virus (%) [37]		0.4			1.6			3.5			7.0			7.0	
*H. pylori* (%) [38]		23.6			47.4			66.1			73.4			72.6	

a Sample size in the National Health and Nutrition Survey in 2007.

b For those aged 20–29 y, the mean (standard error) was 40.4 (2.7) in men (*n* = 324) and 53.9 (2.5) in women (*n* = 395).

c Reported for the total age group of both sexes combined.

PUFA, polyunsaturated fatty acids; SE, standard error; SFA, saturated fatty acids; TFA, trans fatty acids.

We used self-reports to quantify exposures to physical inactivity and alcohol use, while we used measured data for other risk factors. In the physical examination for the 2007 NHNS, a blood test was intended to be conducted more than 4 h after a meal, although a number of blood samples were actually drawn less than 4 h after a meal. Because fasting plasma glucose was the unit for relative risk for high blood glucose adopted in the present study, we applied the following conversion equation proposed by the Committee of the Japan Diabetes Society [29],[30] to predict equivalents of fasting plasma glucose from measurements of hemoglobin A1c:
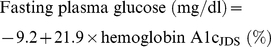
(3)where hemoglobin A1c_JDS_ is a value standardized by calibrators provided by the Japan Diabetes Society and lower than an internationally used value by around 0.4% [29]. As a minor adjustment, we further deducted from this equation a difference in means between predicted fasting plasma glucose and measured casual plasma glucose among 165 participants in the 2007 NHNS who had fasted for more than 8 h (6.4 mg/dl).

In the NHNS, health care professionals measured the blood pressure of seated persons in their right upper arm after 5 min of rest, using a Riva-Rocci mercury manometer. For a trend analysis of cardiovascular mortality attributable to high blood pressure, which is described below, we used the National Nutrition Surveys for 1980–2002 and the NHNS for 2003–2007. These surveys took only one blood pressure measurement per individual until starting to collect two measurements per individual in the 2000 survey. We therefore used a single measurement for the surveys in 1980–1999 and the second measurement for the 2000–2007 surveys. We excluded pregnant or breastfeeding women from the analysis of blood pressure.

For dietary risk exposure variables, dieticians visited households to distribute questionnaires and explain the survey method for diet and lifestyle. Household representatives weighed and recorded the quantity of each food item consumed for one day (excluding holidays). Dieticians visited households again during the survey period to check and correct completed questionnaires. We estimated intakes of dietary trans fatty acids using conversion factors of food items provided by the Cabinet of Japan Food Safety Committee [31]. Considering that nutrition intakes are correlated with energy intake determined by body size, physical activity, and metabolic efficiency, we adjusted intakes of fruit, vegetables, and dietary sodium for total energy intake with a simple linear regression equation having nutrient intake as a dependent variable and total caloric intake as an independent variable [32]. Calorie-adjusted nutrient intakes were computed as the sum of residuals from the regression model and the expected nutrient intake for a person with mean caloric intake.

We used a smoking impact ratio as a more reliable indicator of accumulated exposure to tobacco smoking than the prevalence of current smokers. The smoking impact ratio was defined as total lung cancer mortality in excess of never-smokers in a study population relative to the excess lung cancer mortality among current smokers in a reference population [33],[34]. We used the following formula to calculate smoking impact ratios by age group and sex:
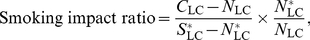
(4)where *C*_LC_ and *N*_LC_ denote lung cancer mortality of the total population and never-smokers, respectively, in a study population (i.e., the Japanese population), and 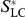 and 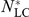 signify lung cancer mortality among current smokers and never-smokers, respectively, in a reference population. We obtained total lung cancer mortality from the redistributed data of vital records described above. Our reference population was residents included in a pooled study of three large-scale cohorts in Japan [15],[35],[36]. Because we also adopted never-smokers' lung cancer mortality in the Japanese population from this pooled study, 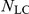 and 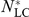 were equivalent to each other in our analysis.

We obtained data on the prevalence of infections with hepatitis B and C viruses and the bacterium *H. pylori* from epidemiological studies undertaken in Japan in the 1990s [37],[38]. Assuming that infection rates do not vary within birth cohorts over time, we applied infection rates by age group in the 1990s to those of corresponding age in 2007. For example, the infection rate for hepatitis B virus in men aged 60–69 y in 2007 was that of men aged 45–54 y in 1991–1993. We considered that all deaths from cervix uteri cancer and adult T-cell lymphoma/leukemia were caused by infections with human papillomavirus and HTLV-1, respectively [26],[39].

In order to measure exposure levels of alcohol use related to deaths from road traffic injuries, we employed a proportion of alcohol-impaired driving, which was defined as driving with breath alcohol concentrations above 0 mg/l, to the total number of cases of road traffic accidents involving four-wheeled vehicles and motorcycles in 2004 (1.7%). We obtained this figure from a past study on alcohol concentrations in the breath of drivers, which used a national dataset prepared by the Japan Institute for Traffic Accident Research and Data Analysis [40].

### Selection of Relative Risks

Tables S1, S2, S3, S4, S5, S6, S7 provide details of relative risks used in this analysis. We conducted a literature review of prospective studies evaluating effects of risk factors on cause-specific deaths in Japan. Strategies for the database search involved contacting authors of key reports and leading experts in the field, and we critically appraised the identified literature. Our motive for undertaking the literature search was to identify evidence from past studies in the Japanese population to be backed up with pooled evidence establishing causalities or associations from the Global Burden of Disease Study [20]. Criteria for the selection of evidence for the Japanese population were: (i) pooled or individual estimates from large-scale prospective observational studies and (ii) confirming causalities or associations that had been already established in past studies. When there was no study for the Japanese population satisfying these conditions, we sought evidence from the Asia-Pacific Cohort Studies Collaboration. If we could not find evidence from this source, then we adopted relative risks identified in the Global Burden of Disease Study. We considered relative risks to be null if they were statistically insignificant. In addition, we had to restrict the source of evidence on relative risks for tobacco smoking to the pooled analysis of large-scale cohorts in Japan, because we used their estimates of current smokers' and never-smokers' lung cancer mortality of a reference population to calculate smoking impact ratios. We excluded mortality from tuberculosis and diabetes mellitus associated with tobacco smoking, because the studies did not examine these causes.

### Counterfactual Distributions of Risk Exposures

As an alternative distribution of risk exposures, we used an optimal distribution in which harmful effects of each risk factor on morbidity and mortality would be minimized in a population (i.e., a theoretical-minimum-risk exposure distribution). With the exception of infections, we obtained information on theoretical-minimum-risk exposure distributions from a previous study in the United States (Table 2) [20].

In the analysis of gains in life expectancy and probabilities of death, we also investigated alternative counterfactual distributions of risk exposures that followed recommendations of clinical guidelines and goals of Health Japan 21. This analysis enabled quantification of potential health gains that would be more realistic than theoretical minimums. We included risk factors in this part of our analysis only if specific control targets were available from these sources and units of measurement corresponded to those of relative risks (Table 2). In order to obtain counterfactual distributions for numerical risks, we used their control threshold as the mean and applied the coefficient of variation to estimate the standard deviation.

The relationship between dietary salt intake and cardiovascular mortality was based on a convincing effect of high dietary salt on systolic blood pressure that was estimated from a meta-analysis of dietary trials (Table S6) [20]. In order to obtain hazards of excess dietary salt intake on cardiovascular death, we first estimated the decrease in systolic blood pressure associated with a reduction in dietary salt intake to individual optimal levels and then applied relative risks of high systolic blood pressure for relevant cardiovascular diseases (Table S1).

### Effects on Life Expectancy and Probabilities of Death

We translated mortality changes into gains in life expectancy at 40 y of age to understand the potential impact of the management of risk factors on longevity. We constructed life tables using observed age-specific mortality rates and mortality that would be expected if risk factor exposures were controlled at alternative levels. We took the differences between these values as showing life expectancy gains that would occur when shifting from an actual risk factor exposure to a counterfactual. We also calculated effects on probabilities of dying between the ages of 15 and 60 y (_45_q_15_) and between 60 and 75 y (_15_q_60_).

### Joint Effects of Multiple Risk Factors for Cardiovascular Mortality

We estimated joint effects of multiple risk factors on excess mortality from cardiovascular diseases and the additional life expectancy at age 40 y that would be achieved under counterfactual distributions. Risk factors included in this part of the analysis were high body mass index, high blood pressure, and high concentrations of blood glucose and low density lipoprotein (LDL) cholesterol. We took account of high dietary sodium intake to compensate for its indirect effect through elevated blood pressure, using the steps described above. We also adopted a 50% reduction of the excess risk of high body mass index on cardiovascular deaths to incorporate a mediation of its associations through other risk factors [21]. We used an additive excess risk scale to correct for correlations of these risk factors and calculate joint relative risks at the individual level. This approach has been described in detail elsewhere [21]. We summed the combined relative risks for individual records to compute population-attributable fractions for the joint effects of these cardiovascular risks.

### Long-Term Trends in Attributable Deaths

To examine contributions of the management of modifiable risk factors to the improvement of life expectancy over time, we estimated the number of deaths from cancers attributable to tobacco smoking and deaths from stroke associated with high blood pressure from 1980 to 2007. We employed the algorithm described above to obtain consistent mortality data throughout this period, from which we used total lung cancer mortality in each year to calculate smoking impact ratios over time. For the analysis of high blood pressure and stroke, we excluded people over 80 y of age because the sample size was insufficient. We also incorporated the above-mentioned mediated effects of dietary sodium intake through raised blood pressure at the individual level.

### Uncertainty Analyses

We conducted statistical simulation to deal with the uncertainty that was introduced by using sample estimates for risk exposures and relative risks [41]. To account for sampling variability, we randomly drew 1,000 sets of values of all components based on samples. In each sequential step of the simulation, we drew for each age–sex group: (i) a random sample of participants in the 2007 NHNS with replacement to obtain the original sample size of those who had no missing value for each risk factor, (ii) a relative risk for each risk–disease pair from a log-normal distribution with means and standard deviations reported in epidemiological studies, (iii) coefficients of the regression of hemoglobin A1c on fasting plasma glucose from a normal distribution with standard deviations that we calculated from information given in a past study (1.0 for the constant term and 0.2 for the coefficient of hemoglobin A1c) [30], (iv) the difference in means between predicted fasting plasma glucose and measured casual plasma glucose in the 2007 NHNS from a normal distribution with mean of 6.4 mg/dl and standard deviation of 1.1 mg/dl that we estimated from the survey data, (v) the proportion of the excess risk of body mass index mediated through systolic blood pressure and fasting plasma glucose from a normal distribution with mean of 0.5 and standard deviation of 0.1 [21], and (vi) lung cancer mortality of current smokers and never-smokers from a normal distribution with means and standard deviations estimated from the pooled analysis of Japanese cohorts [15],[35]. We used each sampled set of risk exposures and relative risks to compute population-attributable fractions, mortality attributable to each risk factor or a combination of risk factors, and changes in life expectancy under counterfactual distributions. We defined a 95% confidence interval (CI) by a span across the estimates of each outcome at the 2.5th and 97.5th percentiles of the 1,000 simulations.

## Results

### Contributions of Health Risks to Cause-Specific Mortality in 2007

Tables S8 and S9 provide population-attributable fractions of the 16 modifiable risk factors and a combination of physiological risk factors for mortality from non-communicable diseases and injuries by age group and sex in 2007. These fractions cannot to be summed across risk factors for a single cause of death, because causal relationships between risk factors are not considered in the analysis of individual risk factors.

Under the theoretically minimum counterfactuals listed in Table 2, tobacco smoking and high blood pressure were the two major single contributors to the number of deaths from non-communicable diseases and injuries (Table 4). Among the total of 960,000 deaths from causes included in this study, tobacco smoking was associated with 129,000 deaths (95% CI: 115,000–154,000). Approximately three-quarters of these deaths occurred in men (95,000 deaths, 95% CI: 88,000–103,000), although the attributable mortality was still substantial for women (34,000 deaths, 95% CI: 23,000–57,000). In men, 70% of deaths attributable to this risk factor were caused by cancers and took place among those aged 45–79 y. In women, cardiovascular diseases and cancers accounted for 42% and 36%, respectively, of the mortality attributable to tobacco smoking. By disease subtypes for sexes combined, lung cancer was the leading cause (42,000 deaths, 95% CI: 39,000–45,000), followed by ischemic heart disease (27,000 deaths, 95% CI: 19,000–42,000) and chronic obstructive pulmonary disease (13,000 deaths, 95% CI: 9,000–16,000).

**Table 4:** The number of deaths attributable to risk factors in Japan, 2007 (in thousands).

Sex, Risk Factor	Total		Cardiovascular		Cancer		Diabetes Mellitus		Respiratory		Other NCD		Injuries	
**Sexes combined**														
High blood glucose	34.1	(26.4, 43.1)	27.2	(19.5, 36.2)			6.9							
High LDL cholesterol	23.9	(16.7, 31.2)	23.9	(16.7, 31.2)										
High blood pressure	103.9	(86.0, 119.1)	103.9	(86.0, 119.1)										
High body mass index	19.0	(16.1, 21.9)	13.8	(11.1, 16.4)	4.1	(3.4, 4.9)	1.1	(0.8, 1.3)						
Alcohol use	30.6	(27.5, 34.7)	−2.0	(−4.0, 0.0)	18.2	(16.2, 20.8)	−0.1	(−0.1, −0.1)			11.6	(10.6, 12.7)	2.9	(1.9, 4.6)
Tobacco smoking	128.9	(115.5, 153.6)	33.4	(25.4, 48.8)	77.4	(72.3, 83.9)			18.1	(12.6, 26.4)				
Physical inactivity	52.2	(46.7, 57.7)	42.2	(36.6, 47.6)	9.3	(8.5, 10.0)	0.7	(0.6, 0.9)						
High TFA intake	0.0	(0.0, 0.0)	0.0	(0.0, 0.0)										
Low PUFA intake	21.2	(8.1, 38.7)	21.2	(8.1, 38.7)										
High dietary sodium intake	34.0	(27.3, 39.4)	19.0	(16.1, 22.3)	14.9	(8.8, 19.6)								
Low fruit and vegetable intake	8.9	(6.7, 10.8)	5.1	(3.3, 6.7)	3.8	(2.5, 4.9)								
Hepatitis B virus	11.6	(9.8, 13.5)			11.6	(9.8, 13.5)								
Hepatitis C virus	23.0	(21.3, 24.5)			23.0	(21.3, 24.5)								
*H. pylori*	30.6	(27.2, 33.5)			30.6	(27.2, 33.5)								
Human papillomavirus	2.6				2.6									
HTLV-1	1.1				1.1									
Joint risk^a^	157.0	(144.0, 173.4)	157.0	(144.0, 173.4)										
**Men**														
High blood glucose	17.2	(12.7, 22.2)	14.3	(9.8, 19.3)			2.9							
High LDL cholesterol	12.2	(8.1, 15.9)	12.2	(8.1, 15.9)										
High blood pressure	50.1	(39.9, 58.5)	50.1	(39.9, 58.5)										
High body mass index	12.1	(10.0, 14.3)	9.6	(7.6, 11.6)	2.0	(1.6, 2.6)	0.5	(0.4, 0.7)						
Alcohol use	25.8	(22.6, 29.5)	−1.7	(−3.7, 0.2)	15.9	(13.7, 18.2)	−0.1	(−0.1, −0.1)			9.0	(8.2, 9.7)	2.8	(1.7, 4.4)
Tobacco smoking	94.9	(87.7, 103.4)	19.3	(15.4, 24.5)	66.5	(61.8, 71.2)			9.1	(6.8, 10.9)				
Physical inactivity	25.9	(22.8, 29.4)	21.0	(18.1, 24.4)	4.6	(4.0, 5.1)	0.3	(0.3, 0.4)						
High TFA intake	0.0	(0.0, 0.0)	0.0	(0.0, 0.0)										
Low PUFA intake	12.0	(5.3, 29.3)	12.0	(5.3, 29.3)										
High dietary sodium intake	18.4	(13.0, 22.7)	8.8	(7.3, 10.2)	9.7	(4.4, 13.7)								
Low fruit and vegetable intake	7.6	(5.5, 9.5)	4.3	(2.5, 5.9)	3.3	(2.1, 4.4)								
Hepatitis B virus	8.0	(6.6, 9.6)			8.0	(6.6, 9.6)								
Hepatitis C virus	14.8	(13.4, 16.1)			14.8	(13.4, 16.1)								
*H. pylori*	20.0	(17.0, 22.4)			20.0	(17.0, 22.4)								
HTLV-1	0.6				0.6									
Joint risk^a^	78.5	(70.8, 87.8)	78.5	(70.8, 87.8)										
**Women**														
High blood glucose	16.9	(11.6, 23.3)	12.9	(7.6, 19.3)			4.0							
High LDL cholesterol	11.7	(6.5, 18.0)	11.7	(6.5, 18.0)										
High blood pressure	53.9	(40.0, 66.9)	53.9	(40.0, 66.9)										
High body mass index	6.8	(5.0, 8.9)	4.2	(2.5, 5.9)	2.1	(1.6, 2.6)	0.5	(0.3, 0.8)						
Alcohol use	4.8	(3.8, 6.3)	−0.3	(−0.5, −0.1)	2.3	(1.7, 3.3)	−0.0	(−0.1, −0.0)			2.6	(2.1, 3.4)	0.2	(0.1, 0.3)
Tobacco smoking	34.0	(22.9, 56.5)	14.1	(7.3, 28.2)	10.9	(8.3, 15.7)			9.0	(4.0, 17.4)				
Physical inactivity	26.3	(21.6, 30.9)	21.2	(16.5, 25.9)	4.7	(4.2, 5.2)	0.4	(0.3, 0.5)						
High TFA intake	0.0	(0.0, 0.0)	0.0	(0.0, 0.0)										
Low PUFA intake	9.3	(4.0, 16.1)	9.3	(4.0, 16.1)										
High dietary sodium intake	15.6	(11.3, 19.2)	10.3	(7.7, 13.1)	5.3	(1.9, 7.5)								
Low fruit and vegetable intake	1.3	(0.9, 1.7)	0.8	(0.4, 1.2)	0.5	(0.3, 0.6)								
Hepatitis B virus	3.6	(2.8, 4.5)			3.6	(2.8, 4.5)								
Hepatitis C virus	8.2	(7.3, 9.0)			8.2	(7.3, 9.0)								
*H. pylori*	10.6	(8.9, 12.0)			10.6	(8.9, 12.0)								
Human papillomavirus	2.6				2.6									
HTLV-1	0.5				0.5									
Joint risk^a^	78.5	(66.9, 91.1)	78.5	(66.9, 91.1)										

Values in parentheses indicate lower and upper bounds of 95% CI.

a A combination of high blood glucose, high LDL cholesterol, high blood pressure (directly, and indirectly through high dietary salt intake), and high body mass index.

NCD, non-communicable disease; PUFA, polyunsaturated fatty acids, TFA, trans fatty acids.

High blood pressure was associated with 104,000 cardiovascular deaths (95% CI: 86,000–119,000) in 2007. This was the greatest risk factor for cardiovascular mortality of all risk factors included in this analysis, and the mortality burden was shared evenly between the sexes. A majority of deaths attributable to high blood pressure occurred among people aged 70 y and over (85,000 deaths) and were caused by stroke (47,000 deaths, 95% CI: 38,000–56,000) or ischemic heart disease (28,000 deaths, 95% CI: 15,000–39,000).

Although the numbers of attributable deaths for other physiological, lifestyle, dietary, and infectious factors were small when compared to those for tobacco smoking and high blood pressure, most of these other factors were associated with tens of thousands of deaths from non-communicable diseases and external causes. Physical inactivity was associated with 52,000 deaths (95% CI: 47,000–58,000), and 75% of them occurred among people aged 70 y and older. Ischemic heart disease was the major cause of mortality attributable to this risk factor (31,000 deaths, 95% CI: 28,000–35,000). High blood glucose was associated with 34,000 deaths (95% CI: 26,000–43,000), of which 75% occurred among people aged 70 y and over and 68% were caused by ischemic heart disease. High dietary salt intake was associated with 19,000 cardiovascular deaths (95% CI: 16,000–22,000), which were included in cardiovascular mortality attributable to high blood pressure, and there were 15,000 deaths from stomach cancer (95% CI: 9,000–20,000). Seventy-six percent of deaths attributable to this risk factor occurred among people aged 70 y and over.

Alcohol use was associated with 31,000 deaths (95% CI: 27,000–35,000) from non-communicable diseases and injuries, 84% of which occurred among men. A major cause of death attributable to this risk factor was liver cirrhosis (11,000 deaths, 95% CI: 10,000–12,000), followed by liver cancer (6,000 deaths, 95% CI: 4,000–8,000), esophagus cancer (5,000 deaths, 95% CI: 4,000–5,000), and colon cancer (4,000 deaths, 95% CI: 4,000–5,000). Alcohol use was associated with 3,000 (95% CI: 2,000–5,000) out of 83,000 deaths of people aged 20 y and over from external causes included in this study. Two thousand deaths were from suicide (95% CI: 1,000–4,000), and there were fewer than 1,000 deaths each attributable to falls, road traffic accidents, homicide, and other injuries. Most of the suicide deaths attributable to alcohol use occurred among men, particularly those aged 30 to 59 y (71%).

Infection with *H. pylori* was associated with 31,000 deaths from gastric cancer in 2007 (95% CI: 27,000–34,000). Seventy-two percent of these deaths occurred among people aged 70 y and older. Infection with hepatitis C virus was associated with 23,000 deaths from liver cancer (95% CI: 21,000–24,000). Forty-five percent of these deaths were in people aged 70–79 y, including those born in the early 1930s. For both *H. pylori* and hepatitis C virus infections, around 65% of the attributable mortality took place in men.

High LDL cholesterol was associated with 24,000 cardiovascular deaths (95% CI: 17,000–31,000), largely from ischemic heart disease (23,000 deaths, 95% CI: 16,000–30,000). Low dietary intake of polyunsaturated fatty acids was associated with 21,000 deaths from ischemic heart disease (95% CI: 8,000–39,000), and 47% of these deaths occurred among people aged 80 y and over. High body mass index was associated with 19,000 deaths (95% CI: 16,000–22,000): 64% of these deaths occurred in men, and ischemic heart disease was the major cause (11,000 deaths, 95% CI: 8,000–13,000).

If systolic blood pressure (directly, and indirectly through dietary salt intake), blood glucose, LDL cholesterol, and body mass index were controlled jointly to their optimal distributions, i.e., theoretical-minimum-risk exposure distributions, 157,000 cardiovascular deaths would have been prevented in 2007 (95% CI: 144,000–173,000). The mortality burden attributable to the combination of these risks was shared equally between the sexes, and a majority of the burden occurred among people aged 70 y and older.

### Effects of Risk Factors on Life Expectancy and Probabilities of Death

**Figure 1:**
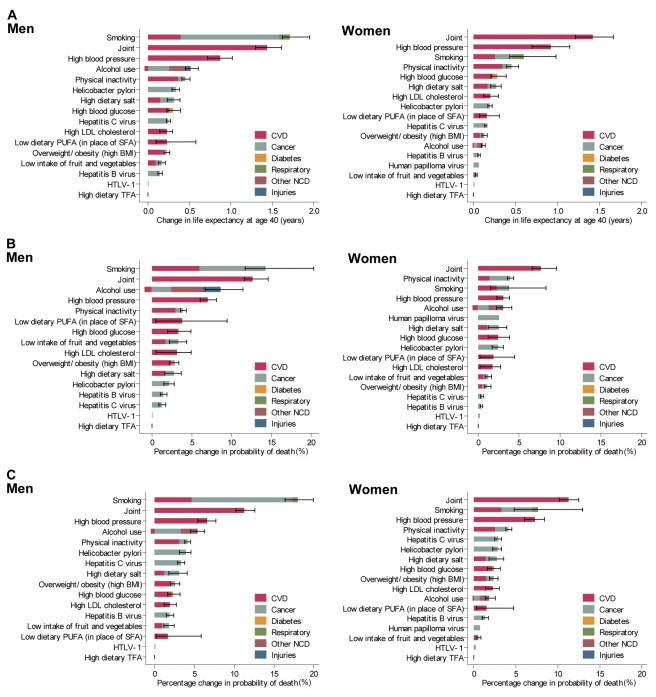
Changes in life expectancy at age 40 y and the probability of death under optimal distributions of risk factors in Japan, 2007. (A) Life expectancy at age 40. (B) Probability of death between 15 and 60 y of age. (C) Probability of death between 60 and 75 y of age. Joint risk is a combination of high blood pressure (directly, or indirectly through high dietary salt intake), high blood glucose, high LDL cholesterol, and high body mass index. BMI, body mass index; CVD, cardiovascular disease; NCD, non-communicable diseases; PUFA, polyunsaturated fatty acids; SFA, saturated fatty acids; TFA, trans fatty acids.

Japanese life expectancy at age 40 y was 40.4 y for men and 46.8 y for women in 2007 [3]. Figure 1 illustrates gains in life expectancy at age 40 y and percentage changes in probabilities of death that would have been expected in 2007 if risk factors had been controlled to their theoretically minimum distributions individually or jointly with others. For men, tobacco smoking was associated with the largest potential increase in life expectancy at age 40 y (1.8 y, 95% CI: 1.6–1.9), followed by a joint effect of multiple physiological factors (1.4 y, 95% CI: 1.3–1.6) and single effects of high systolic blood pressure (0.9 y, 95% CI: 0.7–1.0) and alcohol use (0.5 y, 95% CI: 0.5–0.6). A considerable part of the smoking effect (1.2 y, 95% CI: 1.1–1.3) was accounted for by an expected associated fall in cancer mortality through a decrease in probabilities of dying between the ages of 15 and 60 (45q15) by 8% (95% CI: 7–10) and between the ages of 60 and 75 (15q60) by 13% (95% CI: 12–14). A drop in cardiovascular mortality through the joint control of multiple risks was associated with an expected percentage decrease of 13% (95% CI: 12–15) in 45q15 and 11% (95% CI: 10–13) in 15q60, while controlling high blood pressure was associated with an expected fall of 7% both in 45q15 (95% CI: 6–8) and 15q60 (95% CI: 5–8). Decreasing alcohol use was associated with an expected percentage decrease of 9% (95% CI: 7–11) in 45q15 and 5% (95% CI: 5–6) in 15q60 for men. A substantial part of the potential change in the probability of death among young and middle-aged men through moderate drinking was explained by a fall in mortality from other non-communicable diseases including liver cirrhosis and liver cancer (4%, 95% CI: 4–4) and injuries (2%, 95% CI: 1–4).

For women, controlling systolic blood pressure and tobacco smoking to optimal counterfactuals would have extended life expectancy at age 40 y by 0.9 y (95% CI: 0.7–1.1) and 0.6 y (95% CI: 0.4–1.0), respectively. The impact of tobacco smoking on a probability of death in older women was estimated to be 8% (95% CI: 5–13), which was comparable to that of high blood pressure (7%, 95% CI: 6–8). A joint effect of cardiovascular risk factors on female life expectancy at age 40 y was estimated to be 1.4 y (95% CI: 1.2–1.7), with a decrease in probability of death of 8% (95% CI: 7–10) for younger adults and 11% (95% CI: 10–12) for older ages.

Table 5 shows changes in life expectancy at age 40 y and probabilities of death under the more practical counterfactuals defined by clinical guideline recommendations and national goals. Overall, the gains were less than half of those under theoretically minimum distributions. In both sexes, life expectancy at age 40 y would have increased by 0.7 y (95% CI: 0.6–0.9) through the joint control of cardiovascular risks and by 0.4 y (95% CI: 0.3–0.5) through reducing systolic blood pressure to the distribution recommended by clinical guidelines.

**Table 5:** Changes in life expectancy at age 40 y (e40) and percentage changes in the probability of death between 15 and 60 y of age (45q15) and between 60 and 75 y of age (15q60) under counterfactual distributions of risk factors defined by clinical guidelines and national goals.

Risk Factor	e40 (Years)		45q15 (Percent)		15q60 (Percent)	
**Men**						
High blood glucose	0.1	(0.0, 0.2)	−0.1	(−1.0, −0.1)	−0.7	(−1.2, −0.2)
High LDL cholesterol	0.0	(0.0, 0.0)	−0.6	(−1.3, −0.1)	0.0	(−0.1, 0.0)
High blood pressure	0.4	(0.3, 0.5)	−1.2	(−2.1, −0.3)	−3.0	(−3.7, −2.3)
High body mass index	0.1	(0.1, 0.2)	−1.8	(−2.2, −1.4)	−1.6	(−2.0, −1.2)
High dietary salt intake	0.0	(0.0, 0.0)	−3.4	(−0.2, 0.0)	−2.4	(−0.5, −0.1)
Low fruit and vegetable intake	0.0	(0.0, 0.0)	−0.5	(−0.9, −0.3)	0.0	(0.0, 0.0)
Joint risk^a^	0.7	(0.6, 0.9)	−5.8	(−8.4, −5.1)	−6.0	(−7.4, −5.2)
**Women**						
High blood glucose	0.1	(0.1, 0.2)	−0.2	(−0.9, 0.0)	−0.7	(−1.2, −0.2)
High LDL cholesterol	0.0	(0.0, 0.0)	−0.4	(−0.8, 0.0)	−0.4	(−0.7, −0.3)
High blood pressure	0.4	(0.3, 0.5)	0.0	(0.0, 0.0)	−2.8	(−3.5, −2.1)
High body mass index	0.0	(0.0, 0.1)	−0.1	(−0.4, 0.0)	−1.1	(−1.5, −0.8)
High dietary salt intake	0.0	(0.0, 0.0)	−0.2	(−0.3, −0.1)	−0.4	(−0.5, −0.2)
Low fruit and vegetable intake	0.0	(0.0, 0.0)	0.0	(−0.1, 0.0)	0.0	(0.0, 0.0)
Joint risk^a^	0.7	(0.6, 0.9)	−3.1	(−2.6, −1.0)	−5.6	(−6.6, −4.9)

Values in parentheses indicate lower and upper bounds of 95% CI.

a A combination of high blood glucose, high LDL cholesterol, high blood pressure (directly, and indirectly through high dietary salt intake), and high body mass index.

### Trends in Mortality Attributable to Tobacco Smoking and High Blood Pressure in 1980–2007

**Figure 2:**
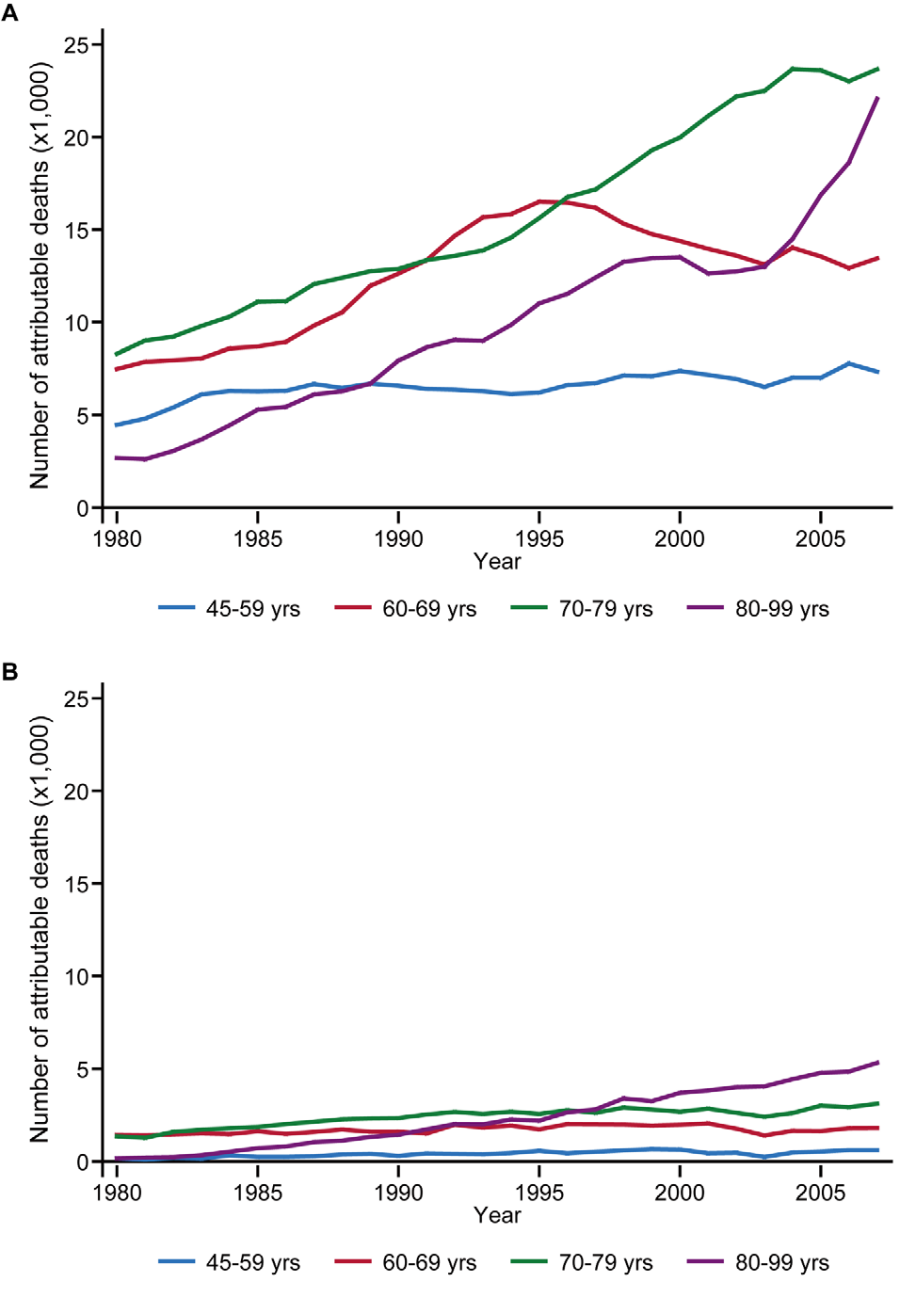
Cancer deaths attributable to tobacco smoking, by age group, 1980–2007. Data for (A) men and (B) women.

Figure 2 illustrates trends in the number of deaths from cancers that were attributable to tobacco smoking from 1980 to 2007. A continuous increase has been observed in men over 70 y old and women over 80 y old. A fall after a peak around 1995 among men aged 60–69 y reflected the fact that the lifetime smoking prevalence reached a peak in the birth cohort of the late 1920s and decreased in the cohort of the late 1930s [42]. This effect manifested again as a peak in attributable cancer mortality around 2005, when the birth cohort of the late 1920s was 70–79 y old. A temporary halt in the increase of cancer deaths attributable to tobacco smoking for men over 80 y old in the early 2000s reflected a reduction in this population group as a result of the 1918 influenza pandemic.

**Figure 3:**
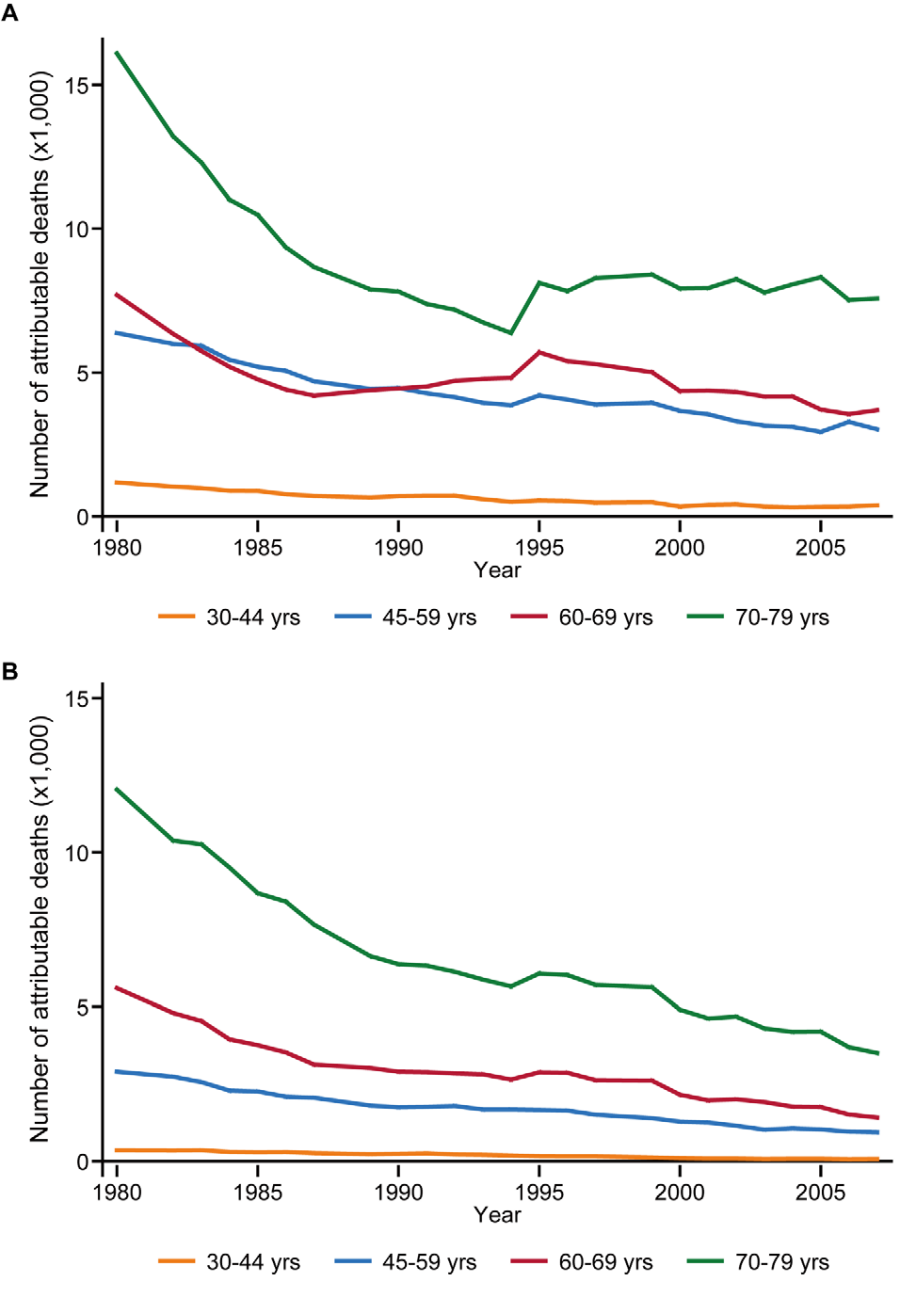
Stroke deaths attributable to high blood pressure, by age group, 1980–2007. Data for (A) men and (B) women.

Figure 3 demonstrates trends in the number of deaths from stroke that were attributable to high blood pressure. Stroke deaths associated with this risk factor, either directly or indirectly through high dietary sodium intake, consistently declined for both sexes under 80 y of age. This favorable trend continued in the 2000s for women and for men under the age of 60 y, but it ceased for elderly men by the mid-1990s.

## Discussion

To our knowledge, this is the first study in Japan to assess and compare effects of a comprehensive list of modifiable risk factors on life expectancy and death from non-communicable diseases and injuries under the framework of comparative risk assessment. Our study indicates that major risks for adult mortality from these causes are tobacco smoking and high blood pressure, as well as a combination of multiple cardiovascular risks. We also demonstrate that, over the past 27 y, cancer mortality attributable to tobacco smoking has increased, especially in the older population, while stroke death associated with high blood pressure has decreased.

The leading single risk factors for adult mortality from non-communicable diseases and injuries in Japan, i.e., tobacco smoking, high blood pressure, physical inactivity, and high blood glucose concentrations, agree with those in the world and the US [2],[20]. The number of deaths attributable to tobacco smoking for Japanese men is large relative to the number attributable to high blood pressure, even compared with the proportion among American men. This result may be related to a substantially higher prevalence of male smokers in Japan than in the US for the past 25 y [43]. Moreover, high body mass index ranks only tenth for both sexes in Japan, while it is one of the top five contributors to mortality in other high- and middle-income countries [2]. This finding reflects the fact that mean body mass index in Japan is low for the income level of the country [44].

Our estimate of the impact of tobacco smoking on male life expectancy at age 40 y (1.8 y) was smaller than those of past cohort studies in Japan. Previous studies showed that, according to smoking status at the time of the baseline survey, life expectancy for men aged 40 y in the total population was shorter than that of never-smokers by around 2.5 y [14],[16]. Use of different exposure measurements may explain part of the difference in estimated impacts of tobacco smoking between the present and past studies. We believe that the smoking impact ratio used in our study is useful for quantifying accumulated smoking risk over a lifetime.

Our results suggest that the threat of tobacco smoking for mortality is enormous in men and has been increasing over time through the accumulation of exposure to this risk in the older population. A previous study showed that lifetime smoking prevalence was low for the generation born in the late 1930s who experienced the deprivation in the early postwar years, but rose thereafter until it peaked for the birth cohort of the 1950s [42]. These findings imply that, without effective policy interventions, the increasing trend in tobacco-associated mortality may continue until at least the late 2030s, when the birth cohort of the late 1950s reaches the age of 80 y. Aiming to decrease the disease burden related to tobacco smoking in the population, the Japanese government enacted the Health Promotion Act in 2002 to support prevention of passive smoking in public places. Based on this legislation, Health Japan 21 specified four targets for tobacco smoking: (i) increasing knowledge of the adverse health effects of smoking, (ii) prohibiting minors from smoking, (iii) strengthening separation of smoking areas in public spaces and the workplace, and (iv) dissemination of smoking cessation programs in all municipalities. A final appraisal of Health Japan 21 concluded that there was improvement for all of these targets [7]. However, the prevalence of smoking in the male working population is still high at around 50%, although it has been gradually declining after the implementation of a series of antismoking policies. Moreover, although Japan ratified the World Health Organization Framework Convention on Tobacco Control in 2004, compliance is lagging behind international standards for smoke-free policies, bans on advertising, health warnings on cigarette packages, and antitobacco mass media campaigns [45]. The retail price of the most popular brand of cigarettes is lower than the average among high-income countries [45]. The recent tobacco tax increase in October 2010 was insufficient to induce smokers to give up purchasing tobacco products. Thus, the progress in tobacco control policies is slow, largely because Japanese society is relatively tolerant of this unhealthy behavior. In order to improve the health of the population, policymakers need to implement further stringent antismoking measures that appropriately assess the health impacts of smoking on non-smokers as well as on the smokers themselves.

Our study suggests that a decrease in population blood pressure partly accounts for a reduced mortality from stroke at least since 1980, although the downward trend leveled off for elderly men in the early 1990s. Stroke mortality started decreasing in the late 1960s and has been the major contributor to the increase of life expectancy in Japan [4]; our finding backs up the idea that a reduction in population blood pressure has contributed to improved longevity. Potential key factors for the decline of blood pressure in the Japanese population may include increased use of blood-pressure-lowering drugs among patients with hypertension, and a reduction in dietary salt intake [46]. These successes may be attributed to the support of the national government for community-based programs for hypertension control that were proven to be effective in pilot studies conducted in the 1960s and 1970s [47]. In 1982, a national act on health and medical care was enacted that required all municipalities to provide residents aged 40 y and over with health screening and educational services for prevention of cardiovascular diseases [47].

Despite the decrease in stroke mortality attributable to high blood pressure, this is still the major risk factor for cardiovascular mortality in Japan. The management of high blood pressure is not adequate even under the practical standards of domestic clinical guidelines. In 2007, less than 60% of hypertensive patients took antihypertensive medication daily, and only 20% had their blood pressure controlled [48]. These treatment coverage and control rates are substantially lower than those in the US [48]. Previous studies pointed out that an acceptance of higher control thresholds of blood pressure among physicians [49] and insufficient treatment regimens [50] might partly explain the poor control of blood pressure with antihypertensive drugs in Japanese clinical practice. A thorough investigation is necessary to understand what makes the quality of care for hypertension so low at the population level, including investigating adherence of patients to prescriptions. Furthermore, lowering dietary sodium intake is crucial for decreasing blood pressure as well as mortality from stomach cancer in Japan. The Japanese diet is traditionally high in salt, which mainly comes from soy sauce, miso soup, and salted vegetables and fish [51]. Although Health Japan 21 was successful in decreasing average daily salt intake from 13.5 g in the baseline year to 10.7 g in 2009 [7], it is well above the global target of 5 g/d set by the World Health Organization [52]. Extra efforts by the industrial sector on ingredient labeling for consumers and reducing sodium content in commercially processed products are essential.

Another key finding of our study is that a considerable number of deaths from cardiovascular diseases would be prevented through joint control of multiple risk factors in Japan. In addition to the traditional approach of focusing on single risk factors, health education and effective treatment based on absolute risk have great potential for improving primary and secondary prevention of cardiovascular mortality. Our results support current domestic efforts to target high-risk populations, such as cardiovascular risk stratification according to categories of multiple risks [53],[54] and the development of risk assessment charts for Japanese people [55].

Our study suggests that physical inactivity contributes to a substantial mortality from non-communicable diseases in Japan. Lack of exercise is common: in 2008, two-thirds of the Japanese adult population engaged in less than 30 min of moderate activity per week or less than 20 min of vigorous activity three times per week [56]. Considering global efforts to promote physical activity for the prevention of non-communicable diseases [57], it is important to strengthen policies for improving public understanding of the role of physical training in disease prevention, and provide support for individual's efforts to start having regular exercise.

Our results suggest that mortality from external causes, such as suicide and traffic accidents, associated with alcohol use is fairly small in Japan. For suicide, relative risks of alcohol use were insignificant, except for heavy drinking, in a large Japanese cohort study [58]. Major reasons for suicide in the male working population are psychiatric disorders and economic reasons such as business failure, unemployment, and debts [59], which suggest that direct risk factors for deaths from suicide are psychosocial, and alcohol use itself may have only an indirect effect. Regarding road traffic accidents, it remains to be seen in future research how robust our result is, because our information on road traffic accidents was limited to published crude estimates on risk exposures and relative risks. We also applied relative risks of suicide to falls, homicide, and other injuries because of the lack of evidence. In order to make a convincing argument on mortality from alcohol-related injuries in Japan, we need to wait for more detailed data and evidence to be accumulated and be made accessible.

One of distinctive characteristics of adult mortality in Japan is a large number of cancer deaths attributable to infectious agents, which is possibly common in East-Asian countries [26],[60]. Mortality from stomach cancer related to *H. pylori* is substantial in Japan, because of the relatively high prevalence of this infection [26]. However, a decline in the prevalence of *H. pylori* infection was observed among people born after 1955 [38], who experienced improved hygienic conditions under rapid economic growth in early childhood. This favorable trend predicts a future reduction in the burden of gastric cancer attributable to *H. pylori* in Japan. Moreover, chronic infection from hepatitis C virus is responsible for the majority of cases of hepatocellular carcinoma in Japan, while hepatitis B virus plays the major role in most Asian countries [61]. A considerable part of mortality attributable to hepatitis C virus infection occurred among people born in the early 1930s. The risk of becoming infected with hepatitis viruses was high in this birth cohort, because intravenous use of methamphetamines was endemic in Japan in their young adulthood [27],[62]. The spread of hepatitis viruses from drug abusers to the general population in the 1950s and 1960s was most likely mediated by transfusion of unscreened or commercial blood and blood products and by medical practices such as needle sharing for immunizations [62]. The decreasing prevalence of infections with hepatitis C virus after the birth cohort of around 1935 indicates that the mortality burden of this infectious agent will diminish in the foreseeable future.

Will the estimated improvements in population health outcomes be worth all the efforts required of the government, citizens, and health care workers involved in the modification of risk factors? The overall increases in life expectancy associated with improved risk factor exposures may appear small in comparison with observed improvements in Japanese longevity over previous decades. This is, however, consistent with a past study's finding showing that even complete elimination of deaths from major causes would not affect life expectancy as much as anticipated in the US, and an additional drop in mortality would have only a marginal effect in countries where the rapid increases of life expectancy have already ended [63]. A study in Sweden also suggested that the main improvements in increasing a life span come from changes in death rates among the oldest groups [64]. In order for the aging population to continue the constant progress in longevity, it is essential to decrease mortality in older ages through the control of risk factors for non-communicable diseases and injuries. Working on risk factors in younger generations is especially important from this standpoint to ensure further improvement in Japanese life expectancy in the long run.

Our study was based on global efforts of various agencies to pool evidence on causality and consistency of relative risks. We also used Japanese population evidence from large-scale cohort studies if they confirmed established causality, although effects of excess risks should not vary across populations [20]. We believe, however, that our effort was justified because the pooled estimates of these large-scale studies reflected the magnitude of the proportional effects of risk factors in the specific context of the Japanese population.

Our analysis had several limitations that should be noted. First, we focused on impacts of risk factors on mortality relative to changes in life expectancy and did not account for morbidity and disability. It is important in future studies to integrate these nonfatal health outcomes and examine disability-adjusted life years under the framework of comparative risk assessment in Japan. This is particularly true because the prognosis of non-communicable diseases has been improving with enhanced access to care, advances in medical technologies, and the standardization of treatment. Second, we could not incorporate standard metabolic equivalents in the categorization of exposures to physical inactivity because of the lack of detailed data from the 2007 survey, but instead we adopted a broader classification based on only the intensity and duration of physical activity that was used in the Global Burden of Disease Study in 2000. Third, data on dietary sodium intake until 1995 were recorded at the household level, which might increase uncertainty concerning the estimated stroke mortality attributable to high blood pressure in the early years. Fourth, we employed LDL cholesterol as an exposure metric for high concentrations of serum cholesterol, because it is the major atherogenic lipoprotein and a primary target for prevention of coronary heart disease [65]. It is, however, also a possibility for future studies to examine effects of low concentrations of high density lipoproteins, because growing evidence indicates that it plays an important role in atherogenesis [65]. Last, some of the Japanese population studies included in this analysis did not exclude disease end points occurring within a certain period after baseline in estimating relative risks. A few studies, however, conducted additional analyses and proved that changes in their results were minor.

To sustain the trend of longevity in Japan for the 21st century, additional efforts in a variety of fields are required for decreasing adult mortality from chronic diseases and injuries. A first step will be to powerfully promote effective programs for smoking cessation. Indeed, tobacco smoking is deeply rooted in Japanese society, and coordinating among interests of ministries and industries is hard. Health care professionals, including physicians, who are highly conscious of the harms of tobacco will play the primary role in treatment of smoking and creating an environment for implementation of stringent tobacco control policies. Moreover, it is urgent to establish a monitoring system for management of high blood pressure at the national level. Further investigation through national health surveys will help understand factors that contribute to the inadequate control of blood pressure in the Japanese population. Measuring the quality of the care that is actually delivered by interventions will be of paramount importance in the assessment of current policies and programs for the treatment of multiple cardiovascular risks including hypertension. These concerted actions in research, public health, clinical practice, and policymaking will be the key for maintaining good population health in the aging society.

## Supporting Information

Alternative Language Article S1 Translation of the article into Japanese by the authors.
(DOCX)

Table S1 Relative risks for the effects of physiological risk factors on non-communicable diseases.
(DOCX)

Table S2 Relative risks for the effects of alcohol use on disease outcomes from meta-analyses.
(DOCX)

Table S3 Relative risks for the effects of alcohol use on disease outcomes from Japanese studies.
(DOCX)

Table S4 Relative risks for the effects of tobacco smoking on disease outcomes.
(DOCX)

Table S5 Relative risks for the effects of physical inactivity on disease outcomes.
(DOCX)

Table S6 Relative risks for the effects of dietary risk factors on disease outcomes.
(DOCX)

Table S7 Relative risks for the effects of infections on disease outcomes.
(DOCX)

Table S8 Population-attributable fractions of cause-specific mortality attributable to individual risk factors in men in 2007.
(DOCX)

Table S9 Population-attributable fractions of cause-specific mortality attributable to individual risk factors in women in 2007.
(DOCX)

## Abbreviations

CI: confidence interval
HTLV-1: human T-lymphotropic virus type 1
LDL: low density lipoprotein
NHNS: National Health and Nutrition Survey
