# Supplementary material for: Adult Mortality Attributable to Preventable Risk Factors for Non-Communicable Diseases and Injuries in Japan: A Comparative Risk Assessment
Source: PLoS Med. 2012 Jan 24;9(1):e1001160. doi: 10.1371/journal.pmed.1001160 (PMC3265534; doi:10.1371/journal.pmed.1001160)
Supplement: Alternative Language Article S1 — Translation of the article into Japanese by the authors. (DOCX) [file pmed.1001160.s001.docx]

**Alternative Language Article S1: Translation of the article into Japanese by authors.**

**日本における予防可能な要因に起因する非感染性疾患及び外傷による成人死亡：比較リスク分析**

池田奈由^1^*、井上真奈美^2^、磯博康^3^、池田俊也^4^、佐藤敏彦^5^、野田光彦^6^、溝上哲也^7^、今野弘規^3^、齋藤英子^1^、片野田耕太^8^、祖父江友孝^8^、津金昌一郎^2^、モーセン・ナガヴィ^9^、マジッド・エザティ^10^、渋谷健司^1^

1. 東京大学大学院医学系研究科国際保健政策学教室
2. 独立行政法人国立がん研究センターがん予防・検診研究センター予防研究部
3. 大阪大学医学部公衆衛生学教室
4. 国際医療福祉大学大学院医療福祉学研究科
5. 北里大学医学部北里臨床研究センター
6. 独立行政法人国立国際医療研究センター病院糖尿病・代謝症候群診療部
7. 独立行政法人国立国際医療研究センター研究所国際保健医療研究部
8. 独立行政法人国立がん研究センターがん対策情報センターがん情報統計部
9. ワシントン大学保健指標評価研究所
10. インペリアル・カレッジ公衆衛生学部疫学・生物統計学教室MRC-HPA環境・保健センター

* 問い合わせ先：ikedan@m.u-tokyo.ac.jp

**要約**

**背景：**日本は世界で最も長い平均寿命を達成したが、国民の健康を更に向上するためには、予防可能な要因に起因する死亡者数に関する整合性があって比較可能なエビデンスに基づき、健康政策やプログラムにおける優先順位を設定する必要がある。先行研究では、日本における個々の要因の影響が検証されたが、非感染性疾患及び外傷における複数の改善可能な要因の影響について、標準的な枠組を利用して比較・検証した研究はまだない。本研究では、16の要因が死因別死亡と平均余命に及ぼす影響を推定・比較した。

**方法と結果：**国民健康・栄養調査および疫学調査から要因への曝露に関するデータを入手した。また、人口動態調査より不明瞭なコードを調整した死因別死亡数を、疫学調査とメタ解析より相対危険度に関する情報を得た。比較リスク評価手法(Comparative Risk Assessment, CRA)を用いて、過剰リスクが死亡と40歳平均余命へ及ぼす影響を推定した。2007年の死亡のうち、喫煙が12.9万人（95%信頼区間[CI]：11.5万–15.4万人）、高血圧が10.4万人（95% CI：8.6万–11.9万人）と関連して最も多く、続いて運動不足（5.2万人、95% CI：4.7万–5.8万人）、高血糖（3.4万人、95% CI：2.6万–4.3万人）、塩分の過剰摂取（3.4万人、95% CI：2.7万–3.9万人）、アルコール摂取（3.1万人、95% CI：2.8万–3.5万人）であった。過去数十年では、喫煙に関連するがん死亡者数が高齢者において増加する一方、高血圧に関連する脳卒中死亡者数は減少傾向にあった。複数の心血管要因が最適水準にとどまっていた場合、2007年の40歳平均余命は男女ともに1.4年（男性、95% CI：1.3–1.6年；女性、95% CI：1.2–1.7年）延伸していたと推定された。

**結論：**喫煙と高血圧は、日本の非感染性疾患及び外傷による成人死亡の主要な要因である。複数の心血管疾患要因が複合的にコントロールされれば、国民の健康に大きな利益をもたらす可能性がある。

**研究の背景と目的**

非感染性疾患及び外傷の要因をコントロールすることは、成人保健の改善において非常に重要である。慢性疾患や外因は全世界の主要な死因であり、2008年には5,700万人の死亡のうちそれぞれ63％、9％を占めた[1]。全世界の死亡の5つの主要な要因は、非感染性疾患に寄与する高血圧、喫煙、高血糖、運動不足、過体重及び肥満であり、これらの因子は有効な介入により改善可能である[2]。この様な状況下では、日本国民において要因を改善することによって予防可能な死亡数に関する整合性があって比較可能なエビデンスに基づく、健康政策やプログラムの優先順位設定が必要である。

日本人の平均寿命は世界一長い。1947年における日本人女性の平均寿命は54.0歳であったが、1986年に81歳と世界一になるまで急速に延び、2009年には86.4歳と過去最高の記録を達成した[3]。こうした継続的な寿命の延長の大部分は、1950年代から60年代前半にかけて小児や若年成人の感染症による死亡率が低下したことと、60年代後半から脳卒中による死亡率が低下したことにより説明される[4]。現在の主要な死因は悪性腫瘍、心疾患、及び脳血管疾患であり、2009年の全死亡数の50％以上を占めた[5]。事故や自殺も過去50年間に亘り上位10位に入っており[5]、特に労働人口における自殺は、1990年代以来の長期に亘る不景気を反映した深刻な社会問題になっている[4]。したがって、日本国民の健康状態を更に改善するには、これらの主要な死因による死亡を防ぐことが極めて重要である。

日本政府は、生活習慣病による早期死亡の予防を通じて国民の健康づくりを図るため、2000年に健康日本21と称される10年間の国民健康増進運動を開始した[6]。この運動では、食事や喫煙、糖尿病などの疾病及び要因の管理を監視するため、59の数値目標が設定された。しかし、結果は必ずしも満足のいくものではなかった。食塩摂取を含む59の指標のうち60％に改善が見られたものの、過体重及び肥満率など残りの40％の指標に関しては悪化もしくは改善無しという結果であった[7]。健康日本21のような国民健康増進運動の成功は、中央及び地方政府が改善可能な要因に起因する疾病負担に関する科学的エビデンスに基づいて、優先順位の高い項目に関する多種多様な活動を調整し資源投資を行うことによるところもあるかもしれない。多くの先行研究で、日本における個別の要因の平均余命への人口寄与割合や影響が数量化されたが[8-16]、単一の包括的な枠組を用いて複数の要因を同時に評価・比較した研究はまだない。

本研究では、日本人成人における非感染性疾患及び外傷による死亡の予防可能な要因を最も包括的に比較評価することを目的とした。比較リスク評価手法を用いて、要因の傷病死亡への寄与を数量化した[17,18]。この標準的で系統的なアプローチは、既に数カ国で主要な要因による疾病および外傷の負担の推計のために適用されている[19-22]。本研究では、要因への曝露と死因別死亡率に関する国レベルのデータと、わが国における大規模前向き研究及びメタ分析からの因果関係に関する疫学的エビデンスを使用し、集団レベルでの死亡及び平均余命の最も重要な要因を特定し、政策立案者が健康政策やプログラムを立案・改訂する上で優先すべき要因の情報を提供する。

**方法**

2007年において要因が最適水準でコントロールされていれば回避できたであろう死亡数を推定した。過度の健康リスクに帰する死亡者数を数量化し比較するために、比較リスク評価手法(Comparative Risk Assessment, CRA)を用いた。この手法は先行研究で詳細に説明されているが[18,20]、要約すると、まず個々の要因の死因別死亡への人口寄与割合を算出した。これは、仮に集団における要因の分布が、実際とは異なるより好ましい理論的最適分布へと転じた場合に達する死亡リスクの減少率を測定するものである。次の方程式を用いて連続値の曝露変数における人口寄与割合を算出した。

人口寄与割合 = $\frac{\int RR\left( x \right)P\left( x \right)ⅆx-\int RR\left( x \right)P_{\left( x \right)}^{'}ⅆx}{\int RR\left( x \right)P\left( x \right)ⅆx}$

*P(x)*及び*P’(x)*はそれぞれ集団における曝露の実際の分布及び理論的最適分布であり、*RR(x)*は曝露レベル*x*における死亡率の相対危険度である。分子の前半と後半部分は、それぞれ実際の分布及び理論的最適分布下での集団における曝露により測られた全死亡リスクを表している。この方法により、各個人の全ての要因への非最適曝露の影響を整合性があって比較可能な方法で算出する事が可能である[21]。複数のカテゴリーで測定された要因に関しては、次の一般化した方程式を用いて人口寄与割合を算出した。

人口寄与割合 = $\frac{\sum_{i=1}^{n} P_{i}\left( RR_{i}-1 \right)}{\sum_{i=1}^{n} P_{i}\left( RR_{i}-1 \right)+1}$

ここでは、*i*は個々のカテゴリーを意味する(*i* = 1, …, n)。

次に、死因別死亡数に人口寄与割合を乗じて、各要因に関連した死因別死亡数を推定した。これらを総計することにより、各要因に起因する死亡数の合計を求めた。しかしながら、各要因の人口寄与割合の推定において要因間の因果関係を考慮していないため、死因別死亡数を複数の要因の間で総計することはできない。

Stata version 11 (StataCorp LP, College Station, Texas, USA)を用いて、全分析を男女別に行った。若年層では非感染性疾患による死亡数がわずかであるため、30歳以上を分析対象とした。ただし、飲酒に起因する外因死亡が相当数に上ると想定されることから20～29歳も対象とした。

**死亡データ**

2007年の人口動態調査から死因別死亡数に関するデータを入手した[23]。死亡診断書において死亡の根底にある原因ではないと想定される不明瞭なコード（例：心停止、心不全、老衰）や不明な原因を再区分するために、Global Burden of Disease 2010 Studyにより開発されたアルゴリズムを応用した[24,25]。この方法によって整合性を確保し、疾病及び関連保健問題の国際統計分類の改訂に基づく変更を解決することで、統計的妥当性や信頼性、比較可能性のある死因別死亡データを得た。

**要因と傷病の選択**

本分析は16の要因を対象とした（表1）。各要因と関連傷病の選択において、先行研究の基準、すなわちi) 質の高い疫学調査から因果関係や関連性に関するエビデンスが利用可能であること、ii) 曝露を改善可能であること、iii) 全国的規模の調査や大規模な集団調査から要因への曝露割合に関するデータが利用可能であることを考慮した[20]。また、B型肝炎ウイルス、C型肝炎ウイルス、ヘリコバクター・ピロリ菌、ヒトパピローマウイルス、ヒトT細胞白血病ウイルス1型への感染は、日本においてがん死亡の重要な要因であることから[26,27]、対象要因に含めた。

**要因曝露の測定とデータソース**

表2は、本分析において使用された要因曝露の測定方法とデータソースのリストであり、表3は2007年における性・年齢階級別の基礎統計を示している。喫煙と感染症、アルコール摂取による交通事故以外に関しては、2007年の国民健康・栄養調査(NHNS)の個票データを使用した。NHNSは国民を代表する標本に基づく調査であり、日本人における健康と栄養状態に関するデータを提供している。この調査には、投薬使用や生活習慣関連要因に関する問診を含む医療従事者による身体状況調査や、自記式質問票による食事と生活様式に関する調査が含まれる[28]。

要因への曝露を数量化するため、運動不足とアルコール摂取については調査回答者による自己申告データを使用し、他の要因については測定データを使用した。2007年度NHNSの健康診断では、食後4時間以上経過後に採血を行う事になっていたが、実際には4時間経過以前に収集された血液サンプルが多かった。本研究で用いた高血糖の相対危険度が空腹時血糖値に対して推定されていたため、日本糖尿病学会(JDS)の委員会が提案した次の換算式[29,30]を用いて、ヘモグロビンA1cから空腹時血糖値に相当する値を推定した。

空腹時血糖値(mg/dl) = -9.2 + 21.9×ヘモグロビンA1c （JDS値）(%)

この際に微調整として、2007年調査で食後8時間以上経過後に採血した165人において測定された随時血糖値の平均値と、この集団で上述の式から推定された空腹時血糖値の平均値との間の差(6.4 mg/dl)を、各調査対象者の空腹時血糖値推定値から差し引くことにより、最終的な空腹時血糖値の推定値として用いた。

NHNSでは、5分の安静の後、座位の患者の右上腕で、医療従事者がリバロッチ水銀血圧計を用いて血圧測定を行った。後述の高血圧に関連する循環器疾患死亡の傾向分析では、1980～2002年度の国民栄養調査と2003～2007年度のNHNSを使用した。血圧の測定回数は、1999年度調査まで1回のみであり、2000年度調査から2回測定されるようになった。したがって、本分析では1980～1999年度調査については単一の測定値、2000～2007年度調査については2回目の測定値を使用した。妊娠中もしくは授乳中の女性は、血圧分析の対象から除外した。

食事については、NHNSでは管理栄養士等の調査員が世帯を訪問して調査票を配布し、栄養摂取に関する調査法を説明した。世帯の代表者が祝祭日を除く1日の摂取品目とその量を測定、記録した。調査期間中に調査員が再び世帯を訪問し、調査票の記入内容のチェックと調査票の回収を行った。本分析では、食品安全委員会による食品中のトランス脂肪酸含有量[31]を用いてトランス脂肪酸摂取量を計算した。また、身体の大きさや運動量、代謝効率で決まるエネルギー摂取量が栄養摂取量と相関関係にあることを考慮し、総カロリー摂取を独立変数とした単回帰方程式を用いて果物・野菜・食事によるナトリウム摂取量を総エネルギー摂取量で調整した[32]。回帰モデルから予測される値と実際の値の間の差と平均エネルギー摂取量における予測栄養摂取量の和を、各調査対象者のエネルギー調整済み栄養摂取量とした[32]。

喫煙への曝露の蓄積に関して、現喫煙者数よりも信頼できる指標としてSmoking impact ratioを採用した。これは、準拠集団における現喫煙者の喫煙未経験者に対する肺がんによる超過死亡率に対する研究対象集団における全人口の喫煙未経験者に対する超過肺がん死亡率の比として定義したものである[33,34]。次の数式を用いて、性・年齢階級別のSmoking impact ratioを算出した。

Smoking impact ratio = $\frac{C_{\mathrm{LC}}-N_{LC}}{{S^{*}}_{LC}-{N^{*}}_{LC}}\times\frac{{N^{*}}_{LC}}{N_{LC}}$

ここで、*C_LC_*及び*N_LC_*はそれぞれ研究対象集団（日本人）における全人口と喫煙未経験者の肺がん死亡率であり、*S***_LC_*及び*N***_LC_*はそれぞれ準拠集団での現喫煙者及び喫煙未経験者における肺がん死亡率を表している。前述の人口動態調査の再区分されたデータから、全肺がん死亡数を入手した。準拠集団は、日本における3つの大規模コホート調査の統合解析の対象者である[15,35,36]。この統合解析データから日本人の喫煙未経験者の肺がん死亡率を採用したため、*N_LC_*及び*N***_LC_*は本分析において同値であった。

B型及びC型肝炎ウイルスとヘリコバクター・ピロリ菌への感染率に関するデータを、1990年代に国内で実施された疫学研究[37,38]より入手した。感染率は出生コホート内で時間の経過と共に変わらないと仮定し、1990年代における年齢階級別感染率を2007年に相当する年齢階級のものとして用いた。例えば、1991～1993年当時45～54歳の男性のB型肝炎ウイルス感染率を、2007年における60～69歳男性の感染率として適用した。子宮頚がんと成人型T細胞白血病による死亡はすべて、それぞれヒトパビローマウイルス及びヒトT細胞白血病ウイルス1型への感染によるものであると見なした[36,39]。

交通事故による死亡に関連するアルコール摂取への曝露レベルとして、2004年における四輪車及び二輪車の全事故件数に対し0mg/Lを超える呼気中アルコール濃度下での運転と定義された飲酒運転の比率（1.7%）を使用した。この数値は、交通事故総合分析センターによる全国データを用いた運転者の呼気中アルコール濃度に関する先行研究から得られた[40]。

**相対リスクの選択**

本分析に用いられた相対リスクの詳細は、付録表S1～S7に掲載されている。日本における要因が死因別死亡に及ぼす影響を評価した前向き研究の文献レビューを行った。データベース検索方法として、主要な文献の著者やその分野における第一人者との連絡も含め、収集した文献を批判的に検討した。文献検索の目的は、Global Burden of Disease Studyが蓄積したエビデンスから因果関係や関連性が立証されているという前提の下で、日本のエビデンスを収集する事にあった[20]。日本のエビデンスの選択基準は、i)個々の大規模前向き観察研究もしくはそれらの統合解析からの推定値であること、ii)先行研究ですでに確立された因果関係もしくは関連性を支持していることの二点であった。これらの条件を満たす日本の研究が無い場合は、アジア太平洋地域におけるコホート共同研究(Asia-Pacific Cohort Studies Collaboration, APCSC)によるエビデンスを検索した。APCSCからエビデンスが見つからなかった場合は、Global Burden of Disease Studyで用いられた値を採用した。統計上有意ではない相対リスクは1とした。さらに、Smoking impact ratio算出の際に日本の大規模コホート統合分析から準拠集団の現喫煙者及び喫煙未経験者の肺がん死亡率を採用したため、喫煙の相対リスクはこの分析による結果を用いた。この統合解析では喫煙に関連した結核と糖尿病による死亡が対象から除外されていたため、本研究においてもこれら二つの死因を喫煙に関する分析の対象から除外した。

**要因への曝露の理論的最適分布**

要因への曝露の実際とは異なる理論的分布として、人口集団において各要因が罹病や死亡に及ぼす有害な影響が最小化される分布（理論的最小リスク曝露分布）を用いた。感染症を除いて、米国での先行研究[20]から理論的最小リスク曝露分布に関する情報を入手した（表2）。

平均余命と死亡確率の変化に関する分析では、健康日本21の目標値と臨床ガイドラインの推奨値を理論的最適分布の平均値として用い、理論上の最小値よりも現実的な潜在的健康利益を検討した。これらの情報源から管理閾値が入手可能であり、その測定単位が相対リスクのものと一致する場合にのみ、分析対象とした（表2）。連続値をとる要因の理論的最適分布を得るため、管理目標値を平均値として変動係数により標準偏差を求めた。

塩分摂取と心血管疾患死亡との関係については、食事療法のメタ解析（付録表S6）から得られた塩分摂取が収縮期血圧に及ぼす影響に関する信憑性のある推定値を基にした[20]。相対リスクを得るため、まず塩分摂取の最適水準までの減少に伴う収縮期血圧の減少分を推定し、それに対して収縮期血圧高値に関連する心血管疾患の相対リスクを適用した（付録表S1）。

**平均余命と死亡確率への影響**

要因の管理が長寿に及ぼす潜在的効果を理解するため、死亡数の変化から40歳平均余命の変化を換算した。要因への曝露が理論的最適分布で管理されていると仮定した場合の死亡数と実際に観察された年齢別の死亡数を用いて生命表を作成した。両者の差をもって、実際の要因の分布から理論的最適分布へと移行する際に生じる平均余命の変化分とした。また、15歳の人が60歳に至るまでの間に死亡する確率(45q15)及び60歳の人が75歳に至るまでの間に死亡する確率(15q60)への影響も推定した。

**心血管死亡に関する複数の要因の複合管理効果**

複数の要因が理論的最適分布に従うことにより達成される心血管疾患死亡率と40歳平均余命への複合効果を推定した。この分析の対象となった要因は、高BMI値、高血圧、高血糖、低LDLコレステロールであった。塩分の過剰摂取が血圧上昇を通じて心血管疾患死亡に及ぼす間接的効果を相殺するため、前述の方法を用いて塩分摂取を考慮した。また、他の要因との交絡性を考慮するため、高BMI値の心血管疾患死亡過剰リスクの半減を採用した[21]。これらの要因の相関を補正し、個人レベルで複合的相対リスクを計算するため、additive excess risk scaleを用いた。このアプローチについては、他の文献にて詳細に記述されている[21]。個人の複合的相対リスクを合計し、心血管疾患要因の複合効果の人口寄与割合を算出した。

**死亡の長期推移**

改善可能な要因の管理に伴う平均余命改善の推移を検討するため、1980～2007年における喫煙に起因するがんおよび高血圧に起因する脳卒中による死亡数を推定した。前述の方法を用いてこの期間における整合性のある死亡データを作成し、各年の肺がん死亡数を用いてSmoking impact ratioを算出した。高血圧に関連する脳卒中死亡推移の分析では、80歳以上は標本の大きさが十分ではない年があったため対象から除外された。また、塩分摂取が血圧上昇を通じてもたらす間接的作用も上述の方法を用いて考慮した。

**信頼区間の推定**

要因への曝露と相対リスクに標本推定値を用いたことから生じる不確実性を考慮するため、統計的シミュレーションを行った[41]。サンプリングによる統計値のばらつきを説明するため、標本に基づいた全ての要素について、1000回の無作為抽出を行った。各回につき、性・年齢階級別にi)2007年NHNSのデータから各要因に欠損値の無い標本を復元抽出、ii)疫学研究から得られた平均と標準偏差を持つ対数正規分布から相対リスクを抽出、iii)先行研究の結果から今回算出した標準偏差を持つ正規分布（切片1.0、ヘモグロビンA1c係数0.2）から空腹時血糖値に対するヘモグロビンA1cの回帰係数を抽出[30]、iv)調査データを用いて推定した平均値6.4mg/dl、標準偏差1.1mg/dlの正規分布から、2007年NHNSの空腹時血糖推定値と随時血糖測定値の平均値の差を抽出、v)平均値0.5、標準偏差0.1である正規分布から、収縮期血圧および空腹時血糖値を通じたBMIの過剰リスク比を抽出[21]、vi)日本人大規模コホート研究の統合解析により推定された平均値と標準偏差を用いた正規分布から、現喫煙者と喫煙未経験者における肺がん死亡率を抽出した[15,35]。以上の要因への曝露と相対リスクの1000回分の抽出データセットを用いて、人口寄与割合、個別もしくは複数の要因に関連する死亡数と平均余命の変化を算出し、それぞれ1000回分の値を得た。また1000回分の値の2.5パーセンタイル値及び97.5パーセンタイル値を、95%信頼区間の下限と上限として用いた。

**結果**

**2007年における要因の死因別死亡への寄与**

付録表S8とS9は、2007年における16の改善可能な要因の個別もしくは複合での非感染性疾患と外傷による死亡への性・年齢階級別人口寄与割合を示している。個別の要因分析において要因間の相関関係を考慮していないため、死因毎に複数の要因の人口寄与割合を合算することはできない。

表2の理論的最適分布の下では、非感染性疾患や外傷による成人死亡に個別で最も大きな影響を与えていた要因は喫煙と高血圧であった（表4）。本研究の対象である死因による死亡96万人のうち、喫煙は12.9万人と関連が認められた（95%信頼区間[CI]：11.5万–15.4万人）。これらの死亡の約75％は男性であったが（9.5万人、95% CI：8.8万–10.3万人）、女性においても相当な数に上った（3.4万人、95% CI：2.3万–5.7万人）。男性の喫煙に関連する死亡の70%はがんによるもので、45～79歳であった。女性の喫煙に関連する死亡の42%が心血管疾患、36%ががんによるものであった。男女計で疾病分類別にみると、最多の死因は肺がんであり（4.2万人、95% CI：3.9万–4.5万人）、虚血性心疾患（2.7万人、95% CI：1.9万–4.2万人）と慢性閉塞性肺疾患（1.3万人、95% CI：0.9万–1.6万人）がそれに続いた。

高血圧は、本分析が対象とする要因のうち最も多くの心血管疾患死亡と関連しており（10.4万人、95% CI：8.6万–11.9万人）、男女で均等であった。これらの死亡の過半数が70歳以上におけるものであり（8.5万人）、脳卒中（4.7万人、95% CI：3.8万–5.6万人）または虚血性心疾患（2.8万人、95% CI：1.5万–3.9万人）によるものであった。

喫煙と高血圧に比べて関連死亡数は少なかったものの、生理学的因子、生活様式、食事、感染症などの他の因子のほとんどが数万人程度の死亡と関連していた。運動不足は5.2万人（95% CI：4.7万–5.8万人）の死亡と関連があり、そのうち75%は70歳以上であり、虚血性心疾患が主な死因であった（3.1万人、95% CI：2.8万–3.5万人）。高血糖は3.4万人（95% CI：2.6万–4.3万人）と関連し、うち75%が70歳以上、68%が虚血性心疾患によるものであった。塩分の過剰摂取は、胃がんによる死亡1.5万人（95% CI：9.0万–2.0万人）と心血管疾患による死亡1.9万人（95% CI：1.6万–2.2万人）と関連していた。塩分の過剰摂取に関連した心血管疾患死亡は、高血圧関連の死亡に含まれる。塩分の過剰摂取に関連した死亡の76％は、70歳以上であった。

アルコール摂取は、非感染性疾患や外傷による死亡3.1万人と関連しており、そのうち84%が男性であった。主な死因は肝硬変（1.1万人、95% CI：1.0万–1.2万人）であり、肝がん（0.6万人、95% CI：0.4万–0.8万人）、食道がん（0.5万人、95% CI：0.4万–0.5万人）、大腸がん（0.4万人、95% CI：0.4万–0.5万人）が続いた。アルコール摂取は、20歳以上の成人の外因による死亡8.3万人のうち0.3万人に関連していた。自殺は0.2万人であり、転落、交通事故、他殺、その他の外傷によるものは0.1万人未満であった。アルコール摂取に関連した自殺のほとんどは男性で、特に30～59歳に多かった（71%）。

ヘリコバクター・ピロリ菌感染は、胃がんによる死亡3.1万人と関連性があり（95% CI：2.7万–3.4万人）、そのうち72%は70歳以上における死亡であった。C型肝炎ウイルス感染は、肝がんによる死亡2.3万人と関連性があり（95% CI：2.1万–2.4万人）、そのうち45%が1930年代初期に生まれた者を含む70～79歳における死亡であった。ヘリコバクター・ピロリ菌およびC型肝炎ウイルスの感染に関連した死亡の約65％が男性における死亡であった。

高LDLコレステロールは、心血管疾患による死亡2.4万人との関連が認められ（95% CI：1.7万–3.1万人）、そのほとんどが虚血性心疾患によるものであった（2.3万人、95% CI：1.6万–3.0万人）。多価不飽和脂肪酸の低摂取は、虚血性心疾患による死亡2.1万人と関連があり（95% CI：0.8万–3.9万人）、そのうち47%が80歳以上であった。高BMI値は、1.9万人の死亡と関連し（95% CI：1.6万–2.2万人）、そのうち64%が男性で、虚血性心疾患が主な死因であった（1.1万人、95% CI：0.8万–1.3万人）。

仮に収縮期血圧（塩分摂取による間接的効果を含む）と血糖値、LDLコレステロール値、BMI値が複合的に最適分布までコントロールされていれば、2007年において心血管疾患による死亡15.7万人が予防可能であったと推定される（95% CI：14.4万–17.3万人）。これらの複合要因に関連する死亡数は男女で均等であり、過半数が70歳以上における死亡であった。

**要因が平均余命と死亡確率へもたらす影響**

2007年の日本人の40歳平均余命は、男性40.4年、女性46.8年であった[3]。図1は、2007年に要因が個別もしくは他の因子と複合で理論的最小リスク分布にコントロールされていた場合の、40歳平均余命と死亡確率の変化を示している。男性の40歳平均余命においては、喫煙に関連した延びが最も大きく（1.8年、95% CI：1.6–1.9年）、複数の生理学的因子の複合的影響（1.4年、95% CI：1.3–1.6年）と高血圧（0.9年、95% CI：0.7–1.0年）、アルコール摂取（0.5年、95% CI：0.5–0.6年）が続いた。喫煙の影響の大部分（1.2年、95% CI：1.1–1.3年）は、がん死亡率の低下に伴う15歳の人が45歳に至るまでの間に死亡する確率（45q15）の8%（95% CI：7–10%）の減少と、60歳の人が75歳に至るまでの間に死亡する確率（15q60）の13%（95% CI：12–14%）の減少より説明された。高血圧のコントロールは、45q15（95% CI：6–8%）及び15q60（95% CI：5–8%）の7％の減少と関連していた一方、複数の要因のコントロールを通じた心血管疾患死亡率の低下は、45q15の13%の減少（95% CI：12–15%）と15q60の11%の減少（95%CI：10–13%）と関連した。男性におけるアルコール摂取の改善は、45q15の9%減（95% CI：7–11%）および15q60の5%減（95% CI：5–6%）と関連した。適量の飲酒にすることによる若年層および中年の男性における死亡確率(45q15)の変化のかなりの部分は、肝硬変や肝がんといった他の非感染性疾患（4%、95% CI：4–4%）および外傷（2%、95% CI：1–4%）による死亡の減少によるものであった。

女性においては、高血圧および喫煙が最適分布でコントロールされていれば、40歳平均余命がそれぞれ0.9年（95% CI：0.7–1.1年）および0.6年（95% CI：0.4–1.0年）延伸していたであろうと推定された。喫煙の15q60への影響は8%と推定され（95% CI：5–13%）、高血圧の7%（95% CI：6–8%）と匹敵するものであった。心血管疾患要因の複合的効果については、45q15の8%減少（95% CI：7–10%）と15q60の11%減少（95% CI：10–12%）に伴う40歳平均余命の延長は1.4年（95% CI：1.2–1.7年）と推定された。

表5は、臨床ガイドラインの推奨値や健康日本21の目標値によって定義されるより現実的な反事実分布を用いた場合の40歳平均余命と死亡確率の変化を示している。全体として、変化は理論的最小リスク分布下での変化の半分以下であった。男女ともに、40歳平均余命は心血管疾患要因の複合的管理により0.7年（95% CI：0.6–0.9年）、収縮期血圧が臨床ガイドラインの推奨値に基づく分布へ低下していれば0.4年（95% CI：0.3–0.5年）上昇していたであろうと推定された。

**1980～2007年の喫煙および高血圧に関連する死亡の推移**

図2は1980～2007年の喫煙に関連するがん死亡数の推移を示している。70歳以上の男性および80歳以上の女性において、継続的な増加傾向が見られた。60～69歳男性における1995年頃のピーク後の減少は、生涯喫煙率が1920年代後半の出生コホートにおいてピークに達した後、1930年代の出生コホートにおいて減少したという事実を反映している[42]。同様の影響は、同群が70～79歳であった2005年前後おいて再びピークとして現れた。2000年代初期に80歳以上の男性における増加傾向が一時的に止まったことは、1918年のインフルエンザの大流行の影響で人口が減少したことを反映している。

図3は高血圧に関連する脳卒中死亡数の推移を示している。高血圧の直接的効果または塩分の過剰摂取を通じた間接的効果に関連した80歳未満の脳卒中死亡者数は、男女ともに減少傾向にあった。2000年代に入ってもこの傾向は男女共に60歳未満で続いているが、高齢男性では1990年半ばまでに終わった。

**考察**

我々の知る限り、本研究は比較リスク評価手法という標準的な枠組を用いて、包括的にリスト化された改善可能な要因が非感染性疾患や外傷による死亡および平均余命へもたらす効果を比較検討した日本で初めての研究である。研究結果から、喫煙と高血圧、複数の心血管疾患リスクの複合効果が、これらの傷病による成人死亡の主要な要因であることが示された。また、過去27年間で高血圧に関連する脳卒中死亡数は減少傾向にある一方、喫煙によるがん死亡数は特に年配層において増加傾向にあることが分かった。

日本における非感染性疾患及び外傷による成人死亡の個別で主要な要因は、喫煙と高血圧、運動不足、高血糖であり、これらは米国や世界におけるものと一致した[2,20]。日本人男性において喫煙に関連する死亡数は高血圧関連の死亡数と比べて多く、この比率は米国人男性のものと比較しても高かった。これは、過去25年間に渡り日本人男性の喫煙率が米国人男性のものに比べてかなり高いことと関係があるかもしれない[43]。さらに、高BMI値は高所得国と中所得国では上位5位内に入ったが[2]、日本では男女共に10位にとどまった。これは、日本の所得レベルに対して平均BMI値が低いことを反映しているものと考えられる[44]。

喫煙が男性の40歳平均余命に及ぼす効果に関する本研究結果（1.8年）は、日本の過去のコホート研究の結果よりも小さかった。先行研究では、男性の40歳平均余命はベースライン調査当時の喫煙未経験者に比べて全体人口の方が2.5年短かった[14,16]。この結果の差の一部は、曝露測定方法の違いによるものもしれない。本研究で用いられたSmoking impact ratioは、生涯に渡り蓄積された喫煙リスクを数量化したものであることから、適切な測定方法であると考えられる。

本研究結果から、男性の喫煙による死亡の脅威は非常に大きく、年齢に伴う曝露の蓄積で増加傾向にあることが示された。先行研究から、生涯喫煙率は1930年代後半に出生し終戦直後の貧困を経験した世代で一時的に減少した後、1950年代の出生コホートまで上昇を続けたことが示されている[42]。以上の結果から、有効な政策介入が行われなければ、喫煙に関連した死亡の増加傾向は、少なくとも1950年代後半の出生コホートが80歳を迎える2030年代まで続く可能性があると考えられる。喫煙による国民の疾病負担の減少を目的として、日本政府は2002年に健康増進法を制定し、公共の場における受動喫煙の防止を推進している。この法律に基づく健康日本21では、喫煙に関して次の4項目の目標を明記した。i)喫煙が健康へ及ぼす悪影響に関する知識の向上、ii)未成年の喫煙防止、iii)公共の場や職場における分煙の強化、iv)全地方自治体における禁煙プログラムの普及である。健康日本21の最終評価では、これらの目標すべてにおいて改善が見られた[7]。しかし、男性の労働人口における喫煙率は一連の禁煙政策の実施に伴い徐々に低下傾向にあるものの、依然として50%前後と高い。加えて、日本は「たばこの規制に関する世界保健機関枠組条約」を2004年に批准したが、禁煙政策、広告の禁止、タバコの包装への健康被害警告の表示、マスメディアの反喫煙キャンペーンへのコンプライアンスは国際水準から遅れを取っている[45]。日本で最も人気の高いタバコ銘柄の定価は、高所得国の平均よりも低い[45]。2010年10月にタバコ税が増税されたが、喫煙者がタバコ製品の購入をあきらめるには不十分であった。このようにタバコ規制政策の進捗は遅く、これは主として日本社会が喫煙という不健康な行動に対して比較的寛容であることを反映していると考えられる。国民の健康を改善するためには、政策立案者は喫煙が喫煙者自身のみならず非喫煙者の健康に及ぼす影響を的確に評価した一層厳格な禁煙対策を実施する必要がある。

本研究結果から、高血圧に関連する脳卒中死亡者数は少なくとも1980年以降減少傾向にあり、高齢男性では1990年代初めから横ばいになったものの、この減少傾向の一部は国民全体の血圧が低下していることに関係していると考えられる。1960年代後半以降の脳卒中死亡率の低下は日本人の平均余命の上昇の主な要因となっていることから[4]、国民全体の血圧の低下が長寿傾向に寄与したという主張を支持している。国民全体の血圧の低下の主な関連要因として、降圧剤服用率の上昇や塩分摂取の減少などが考えられる[46]。これらの成功は、1960年代から70年代に実施されたパイロット研究において効果が証明された地域社会に拠点を置く高血圧管理プログラムを中央政府が支援したことに関連しているかもしれない[47]。1982年に制定された老人保健法では、40歳以上の住民に対し健診や心血管疾患防止のための教育サービスを提供することが全地方自治体に義務付けられた[47]。

高血圧に関連した脳卒中の死亡は減少したものの、高血圧は依然として日本における心血管疾患死亡の主な要因である。国内の臨床ガイドラインにおける実践的な管理推奨値の下でさえ、国民の血圧管理は十分ではない。2007年に降圧剤を毎日服用している高血圧患者は60%に満たず、血圧がコントロールできていたのは20%にすぎなかった[48]。米国と比べて、服用率及び管理率は圧倒的に低い[48]。先行研究では、医師が血圧の高い閾値で許容していること[49]や処方計画が不十分であること[50]が、日本の臨床現場において降圧剤による血圧管理が不十分であることの原因の一つとして指摘された。国民レベルでの高血圧治療の質がなぜそれほど低いのかを理解するためには、患者側の治療の遵守も含めた徹底的な調査が必要である。さらに日本では、塩分摂取を減らすことは、血圧ならびに胃がん死亡率を下げるために極めて重要である。日本食は伝統的に塩分が多く、主に醤油、味噌汁、漬物、塩漬けの魚などから摂取される[51]。健康日本21では、平均一日当たり食塩摂取量を基準年の13.5gから2009年の10.7gまで減少させることに成功したが[7]、世界保健機関が設定した1日5gという国際目標値よりもはるかに高い[52]。消費者のための成分表示や市販の加工食品のナトリウム含有量の低減など、産業界の一層の努力が必要不可欠である。

本研究におけるもう一つの主要な結果は、日本において複数の要因を複合管理することによってかなりの心血管疾患死亡を防ぐことが可能であろうということである。個別の要因に着目した従来型のアプローチに加え、絶対リスクに基づいた健康教育および効果的な治療法を実施することは、心血管疾患死亡の一次・二次予防の改善をもたらす可能性が大きい。この結果は、日本人を対象とした複数の要因区分による心血管疾患リスク層別化[53,54]やリスク評価表の開発[55]といった、高リスク集団をターゲットとした現在の国内における取組を支持している。

本研究の結果から、日本において運動不足が非感染性疾患による死亡に大きく寄与していることが示された。2008年には成人人口の3分の2が、週当たり合計30分の適度な運動か週三回の一回当たり20分の激しい運動、あるいはそれ未満の運動しかしておらず[56]、日本では運動不足が一般的な現象になっている。国際的に非感染性疾患防止のための身体活動の促進への取組が進んでいることから[57]、国民が身体活動の疾病予防に果たす役割を理解し定期的な運動を始めることを支援する環境を改善するための政策の強化が重要であると考えられる。

今回の研究から、アルコール摂取に関連した外因による死亡は、日本ではかなり少ないことが示唆された。これは、男性労働人口における自殺の主な動機が、精神疾患や事業の失敗、失業、借金などの経済的問題であるということを一部反映しているかもしれない[58]。日本の先行研究では、自殺に有意な関連性があったのは大量飲酒のみであることが示されている[59]。しかし、本研究で用いられた交通事故に関する情報は限られており、要因への曝露や相対リスクはおよその公表値であり、転落、他殺、その他の外傷に関しては先行研究がなく自殺の相対リスクを代替として用いたため、本研究結果の頑健性については将来研究において確認する必要がある。日本におけるアルコール摂取に関連した外傷による死亡に関してより説得力のある議論をするためには、より詳細なデータと研究結果が蓄積されて入手しやすくなることを待つ必要がある。

日本における成人死亡の特色の一つとして、感染症に関連するがん死亡が多いことが挙げられるが、これはおそらく東アジアの国々に共通していると考えられる[26,60]。日本ではヘリコバクター・ピロリ菌の感染率が比較的高く[26]、この感染に関連する胃がん死亡は日本において相当数に及んでいる。しかし、1955年以降に出生し幼少期に高度経済成長下での公衆衛生の改善を経験した集団において、ヘリコバクター・ピロリ菌の感染率は低減している [55]。この傾向から、日本のヘリコバクター・ピロリ菌感染に関連した胃がんによる死亡は減少していくことが予測される。さらに、B型肝炎ウイルスはほとんどのアジアの国々で肝細胞癌の主因である一方、日本においては、C型肝炎ウイルス感染が大多数の肝細胞癌の原因となっている[61]。C型肝炎ウイルス感染に関連した死亡のかなりの部分は、1930年代前半に出生した集団におけるものであった。この出生コホートは、青年期に静脈注射による覚醒剤中毒が日本で流行していたことから、肝炎ウイルスに感染するリスクが高かった[27,62]。1950年代および60年代における薬物乱用者から一般人口集団への肝炎ウイルスのまん延は、未検査の血液や市販血液および血液製剤の輸血や予防接種の注射針の使い回しなどの医療行為を介したものである可能性が最も高かった[62]。1935年前後の出生コホート以降、C型肝炎ウイルスの感染率が低下したことから、この感染に関連した死亡は近い将来減少すると考えられる。

本研究で推定された国民の健康の改善は、要因の改善に携わる政府・国民・医療従事者に課せられた努力に見合うものであろうか。過去数十年間で観察された日本人の寿命の変化と比べれば、要因曝露の改善に関連する平均余命の上昇は小さく思えるかもしれない。しかしながら、米国において主な死因による死亡が完全に消滅しても期待されるほどの平均余命への効果が無かったことや、平均余命の急速な延伸が既に終っている国においては死亡数の追加的な減少の効果はごく小さいことといった先行研究の結果と一致している[63]。スウェーデンでの研究からも、寿命延伸は主に最高年齢群における死亡率の変化によるものであることが示唆されている[64]。高齢化社会において継続的に平均余命を改善していくためには、非感染性疾患および外傷の要因のコントロールを通じて高齢者層での死亡率を低下させることが必要不可欠である。この観点から、長期的に日本の平均余命の更なる上昇を図るためには、若年層のうちから要因の改善に取り組むことが特に重要であると考えられる。

本研究は、因果関係に関するエビデンスを蓄積し相対リスクの整合性を保つための様々な機関による国際的努力に基づいている。また、過剰リスクの影響は人口集団間で異ならないはずではあるが、日本の大規模コホート研究において因果関係が確立されている場合は日本のエビデンスを利用した[20]。この点に関しては、少なくとも複数の大規模研究からの統合解析結果であり、日本国民における要因の比例的効果を反映していることから正当化されると考えられる。

本研究には、言及すべきいくつかの制約がある。第一に、平均余命の変化に関連して要因が死亡に与える影響に焦点を当てたが、罹病や障害に関しては考慮しなかったことである。日本の将来の研究において、比較リスク評価手法の枠組の下で非致死的健康アウトカムも統合し、障害調整生存年を検討することが重要である。このことは、治療アクセスの改善や医療技術の進歩、治療の標準化に伴い非感染性疾患の予後が改善してきたことから、特に重要である。第二に、2007年のNHNSにおいて詳細なデータがなかったため、運動不足への曝露の分類において標準的な代謝当量を組み入れることができなかったことである。その代わりに2000年のGlobal Burden of Disease Studyで使用された身体活動の強度と時間のみに基づくより幅広い区分を適用した。第三に、国民栄養調査では1995年まで塩分摂取を始めとする栄養摂取データが世帯レベルで記録されていたため、高血圧に関連する脳卒中死亡数の推定値は、分析対象期間の初期において不確定さが増大している可能性があることである。第四に、LDLコレステロールは主要なアテローム性リポ蛋白質であり冠動脈性心疾患予防の主なターゲットであるため、本研究ではLDLコレステロールを高血清コレステロール濃度への曝露の測度として使用した[65]。しかし、アテローム発生において高HDLコレステロールが重要な役割をしていることを示唆するエビデンスが増えていることから、低HDLコレステロールの影響を検討することも将来の研究における一つの可能性として考えられる[65]。最後に、本分析に活用された日本の研究の中は、相対リスク推定の際にベースライン後の一定期間に発生する疾患エンドポイントを除外していないものがあった。しかし、追加分析を行ったいくつかの研究から、結果の変化は小さいことが示されている。

21世紀において日本人の長寿化の傾向を維持していくためには、様々な方面から慢性疾患や外傷による成人死亡の防止への追加的努力を行っていくことが必要である。最初の第一歩は、効果的な禁煙プログラムを強力に推進することである。実際、喫煙は日本社会に深く根差しており、省庁や産業間の利害関係の調整は難しい。タバコの危害に関して意識の高い医師を含む医療従事者が、喫煙の治療や一層厳しいタバコ規制政策実施のための環境づくりにおいて中心的役割を担うことが求められる。さらに、国レベルでの高血圧管理をモニタリングするシステムの確立が急務である。国民レベルの健康調査を通じた更なる研究は、国民の血圧管理が不十分であることの要因を理解する上での助けとなるであろう。治療介入により実際に達成された効果や質を測定することは、高血圧を含めた複数の心血管疾患リスクの治療に関する現在の政策やプログラムの評価において極めて重要である。こうした研究、公衆衛生、臨床診療、政策立案における共同的な行動は、高齢化社会における国民の健康の維持に不可欠であると考えられる。

**謝辞**

本稿は、平成22‐23年度厚生労働科学研究費補助金（政策科学総合研究事業（政策科学研究推進事業））「我が国の保健医療制度に関する包括的実証研究」（課題番号H22‐政策‐指定‐033）と平成22‐23年度独立行政法人日本学術振興会科学研究費助成事業（科学研究費補助金（基盤研究（B）））「危険因子介入と疾病負担予防に関する包括的評価」（課題番号22390130）の研究結果に基づくものである。

本研究への有益なコメントを頂いたグッダーズ・ダナエイ先生（ハーバード大学研究員）、橋本英樹先生（東京大学教授）、森臨太郎先生（東京大学准教授）、クリストファー・JL・マレー先生（ワシントン大学教授）、大島明先生（大阪府立成人病センター）に深く感謝申し上げます。また、文献とデータの収集、あるいは解析に関する技術的支援を頂いたアマルジャルガル・ダグワドルジ氏（東京大学）、ファーシャド・ファーザドファー氏（ハーバード大学）、李菁華氏（東京大学）、メンルー・ワン氏（ワシントン大学）、事務的支援を頂いた東京大学大学院医学系研究科国際保健政策学教室の近藤一恵氏、佐野留理子氏、佐藤靖子氏、渋谷節子氏に厚く御礼申し上げます。

**図**

図1．2007年の日本における非感染性疾患と外傷の要因の理論的最適分布下における40歳平均余命と死亡確率の変化

A) 40歳平均余命

B) 15歳の人が60歳に至るまでに死亡する確率

C) 60歳の人が75歳に至るまでに死亡する確率

略語：BMI(body mass index)、CVD（心血管疾患）、HTLV-1（ヒトT細胞白血病ウイルス）、NCD（非感染性疾患）、PUFA（多価不飽和脂肪酸）、SFA（飽和脂肪酸）、TFA（トランス脂肪酸）

複合型リスクは、高血圧（塩分摂取による間接的な効果を含む）、高血糖、高LDLコレステロール、及び高BMIを統合したものである。

図2. 1980～2007年における喫煙に関連する年齢別がん死亡数

A) 男性

B) 女性

図3. 1980～2007年における高血圧に関連する年齢別脳卒中死亡数

A) 男性

B) 女性

**表1. 本研究対象の要因および傷病**

**要因、傷病**

*高血糖*

IHD、脳卒中、糖尿病

*高LDLコレステロール*

IHD、脳梗塞

*高血圧*

IHD、脳卒中、高血圧性疾患、その他の心血管疾患^a^

*過体重・肥満*

IHD、脳梗塞、高血圧性疾患、閉経後乳がん、大腸がん、子宮体がん、腎がん、膵がん、糖尿病

*アルコール摂取*

IHD、脳梗塞、脳出血、高血圧性疾患、不整脈、乳がん、結腸直腸がん、食道がん、口腔がん、肝がん、喉頭がん、咽頭がん、その他器官におけるがん^b^、糖尿病、肝硬変、急性および慢性膵炎、交通事故による負傷、転落、他殺および自殺、その他の外傷

*喫煙*

IHD、脳卒中、大動脈瘤および解離、糖尿病、肺がん、食道がん、口腔がん、咽頭がん、胃がん、肝がん、膵がん、子宮頸がん、膀胱がん、腎がん、その他泌尿器がん、白血病、慢性閉塞性肺疾患、下気道感染、喘息、結核

*運動不足*

IHD、脳梗塞、乳がん、大腸がん、糖尿病

*トランス脂肪酸の高摂取*

IHD

*多価不飽和脂肪酸の低摂取*

IHD

*塩分の高摂取*

IHD、脳卒中、高血圧性疾患、その他心血管疾患^a^、胃がん

*果物・野菜の低摂取*

IHD、脳梗塞、結腸直腸がん、食道がん、肺がん、口腔がん、咽頭がん、胃がん

*B型肝炎ウイルス感染*

肝がん

*C型肝炎ウイルス感染*

肝がん

*ヘリコバクター・ピロリ菌感染*

胃がん

*ヒトパピローマウイルス感染*

子宮頸がん

*ヒトT細胞白血病ウイルス1型感染*

成人型T細胞白血病

略語：IHD (ischemic heart disease)　虚血性心疾患

a このカテゴリーには、リウマチ性心疾患、心内膜炎、心筋症、大動脈瘤、末梢血管障害, および他の明確に定義されていない心血管疾患が含まれる。

b このカテゴリーには、ICD-10コードのD00–D24（D09.9を除く）、D26–D37（D37.9を除く）、D38–D48（D38.6、D39.9、D40.9、D41.9、D48.9を除く）が含まれる。

**表2. 要因への曝露の測定方法、データの出所、及び代替分布**

**要因、測定方法, データの出所a 最適分布 ガイドライン・**

**国家目標値**

*高血糖*

空腹時血漿グルコース(mmol/L) 4.9 (0.3) 5.6 (0.3) [66]

*高LDLコレステロール*

LDLコレステロール(mmol/L) 2.0 (0.4) 3.1 (0.7) [54]

*高血圧*

収縮期血圧(mmHg) 115 (6) 130 (7) [53]

*過体重・肥満*

BMI値 (kg/m^2^) 21 (1) 22 (1) [67]

*アルコール摂取*

現在のアルコール摂取量及びパターン 摂取なし^b^

飲酒運転による交通事故

全国交通事故データ, 2004年 [40]

*喫煙*

Smoking impact ratio 喫煙なし

人口動態統計、2007年 [23-25]、統合コホート解析[15,35,36]

*運動不足*

身体活動の強度 高強度

*トランス脂肪酸の高摂取*

総エネルギー比率（%） 0.5 (0.05)

*多価不飽和脂肪酸の低摂取*

総エネルギー比率（%） 10 (1)

*塩分の高摂取*

総カロリー補正済み摂取量（g/日） 0.5 (0.05) 10 (1) [7]

*果物・野菜の低摂取*

総カロリー補正済み摂取量（g/日） 600 (50) 350 (29) [7]

*B型肝炎ウイルス感染*

B型肝炎ウイルス表面抗原の血清反応陽性 感染なし

献血者コホート、1991～1993年[37]

*C型肝炎ウイルス感染*

C型肝炎抗体の血清反応陽性 感染なし

献血者コホート、1991～1993年[37]

*ヘリコバクター・ピロリ菌感染*

抗ヘリコバクター・ピロリ菌免疫グロブリンG抗体の血清反応陽性

多施設研究, 1990年代後半 [38] 感染なし

数値は平均値（標準偏差）である。

^a^ 特別に明記しない限り、2007年国民健康栄養調査[28]より曝露データを入手した。

^b^先行研究では「時々飲酒する」を基準カテゴリーとして相対リスクが推定されていたため、本分析においてもこれを肝がんや自殺における最適区分とした。

**表3．2007年における性・年齢階級別の要因曝露**

**性別、要因**

*男性*

空腹時血糖値

LDLコレステロール

収縮期血圧

BMI

トランス脂肪酸摂取量（総エネルギー比率）

多価不飽和脂肪酸摂取量（総エネルギー比率）

飽和脂肪酸摂取量（総エネルギー比率）

塩分摂取量

果物・野菜の摂取量

飲酒未経験またはかつての飲酒者(%)^b^

飲酒運転による事故・四輪車の交通事故、2004年(%) [40]^c^

高強度の身体活動の実施

喫煙未経験者または元喫煙者

Smoking impact ratio

B型肝炎ウイルス感染(%) [37]

C型肝炎ウイルス感染(%) [37]

ヘリコバクター・ピロリ菌感染(%) [38]

女性

以下、男性のリストに同じ

略語：SE (standard error) 標準誤差

a 2007年国民健康栄養調査におけるサンプルサイズ

b 20～29歳の年齢群における平均値（標準誤差）、男性(n=324)で40.4(2.7)、女性(n=395)で53.9(2.5)であった。

c 性・年齢階級の総計である。

**表4. 2007年日本における要因の関連死亡者数（単位:千人）**

**性別、要因　合計　 心血管疾患　　がん　　糖尿病　　呼吸器系疾患　　その他非感染性疾患　　外傷**

男女計

高血糖

高LDLコレステロール

高血圧

高BMI

アルコール摂取

喫煙

運動不足

トランス脂肪酸の高摂取

多価不飽和脂肪酸の低摂取

塩分の高摂取

果物・野菜の低摂取

B型肝炎ウイルス感染

C型肝炎ウイルス感染

ヘリコバクター・ピロリ菌感染

ヒトパピローマウイルス感染

ヒトT細胞白血病ウイルス1型感染

複合リスクa

男性

以下、上記に同じ（ヒトパピローマウイルス感染を除く）

女性

以下、上記に同じ

^a^高血糖、高LDLコレステロール、高血圧（塩分の高摂取による間接的効果を含む)、高BMI値の複合型

**表5. 臨床ガイドライン推奨値や国家目標値に基づく反事実分布下における40歳平均余命(e40)および15～60歳死亡確率(45q15)と60～75歳死亡確率(15q60)の変化**

**要因　　　　　　　　 e40 (年) 45q15 (%) 15q60 (%)**

男性

高血糖

高LDLコレステロール

高血圧

高BMI

塩分の高摂取

果物・野菜の低摂取

複合リスク^a^

女性

以下、上記に同じ

^a^高血糖、高LDLコレステロール、高血圧（食塩の高摂取による間接的効果を含む)、高BMI値の複合型
