# Supplementary material for: Adult Mortality Attributable to Preventable Risk Factors for Non-Communicable Diseases and Injuries in Japan: A Comparative Risk Assessment
Source: PLoS Med. 2012 Jan 24;9(1):e1001160. doi: 10.1371/journal.pmed.1001160 (PMC3265534; doi:10.1371/journal.pmed.1001160)
Supplement: Table S1 — Relative risks for the effects of physiological risk factors on non-communicable diseases. (DOCX) [file pmed.1001160.s002.docx]

**Table S1: Relative risks for the effects of physiological risk factors on non-communicable diseases.**

| **Risk factor, disease** | **Sex** | **Age (years)** | | | | |
| --- | --- | --- | --- | --- | --- | --- |
|  |  | **30–44** | **45–59** | **60–69** | **70–79** | **≥80** |
| *High blood glucose (per mmol/L increase)* |  |  |  |  |  |  |
| Ischemic heart disease [[1](#_ENREF_1)] | Both | 1.42 | 1.42 | 1.20 | 1.20 | 1.20 |
| Total stroke [[1](#_ENREF_1)] | Both | 1.36 | 1.36 | 1.28 | 1.08 ^b^ | 1.08 ^b^ |
| *High LDL cholesterol (per mmol/L increase)* |  |  |  |  |  |  |
| Ischemic heart disease [[2](#_ENREF_2)] ^a^ | Both | 1.58 ^b^ | 1.37 | 1.19 | 1.19 | 1.12 |
| Ischemic stroke [[3](#_ENREF_3),[4](#_ENREF_4)] | Both | 1.29 | 1.29 | 1.18 | 1.00 ^c^ | 1.00 ^c^ |
| *High blood pressure (per mmHg increase)* |  |  |  |  |  |  |
| Ischemic heart disease [[5](#_ENREF_5)] | Men | 1.02 | 1.02 | 1.01 | 1.00 ^b^ | 1.00 ^b^ |
|  | Women | 1.04 | 1.02 | 1.02 | 1.01 | 1.01 |
| Total stroke [[5](#_ENREF_5)] | Men | 1.03 | 1.03 | 1.02 | 1.02 | 1.02 |
|  | Women | 1.03 | 1.02 | 1.02 | 1.01 | 1.01 |
| Hypertensive disease [[6](#_ENREF_6)] ^a^ | Both | 1.13 | 1.10 | 1.10 | 1.07 | 1.05 |
| Other selected CVD [[6](#_ENREF_6)] ^a^ | Both | 1.06 | 1.05 | 1.05 | 1.03 | 1.03 |
| *High body mass index (per kg/m^2^ increase)* |  |  |  |  |  |  |
| Ischemic heart disease [[3](#_ENREF_3),[7](#_ENREF_7)] | Both | 1.14 | 1.09 | 1.08 | 1.05 | 1.02 |
| Ischemic stroke [[3](#_ENREF_3),[7](#_ENREF_7)] | Both | 1.14 | 1.10 | 1.08 | 1.05 | 1.03 ^b^ |
| Hypertensive disease [[3](#_ENREF_3),[7](#_ENREF_7)] | Both | 1.22 ^b^ | 1.18 ^b^ | 1.14 | 1.11 | 1.08 ^b^ |
| Postmenopausal breast cancer [[8](#_ENREF_8)] | Women |  | 1.04 | 1.04 | 1.04 | 1.04 |
| Colon cancer [[9](#_ENREF_9)] | Men | 1.04 | 1.04 | 1.04 | 1.04 | 1.04 |
|  | Women | 1.03 | 1.03 | 1.03 | 1.03 | 1.03 |
| Corpus uteri cancer [[3](#_ENREF_3),[10](#_ENREF_10)] | Women | 1.10 | 1.10 | 1.10 | 1.10 | 1.10 |
| Kidney cancer [[3](#_ENREF_3),[10](#_ENREF_10)] | Men | 1.04 | 1.04 | 1.04 | 1.04 | 1.04 |
|  | Women | 1.06 | 1.06 | 1.06 | 1.06 | 1.06 |
| Pancreatic cancer [[3](#_ENREF_3),[10](#_ENREF_10)] | Men | 1.01 ^b^ | 1.01 ^b^ | 1.01 ^b^ | 1.01 ^b^ | 1.01 ^b^ |
|  | Women | 1.02 | 1.02 | 1.02 | 1.02 | 1.02 |
| Diabetes mellitus [[3](#_ENREF_3),[11](#_ENREF_11)] | Both | 1.20 | 1.20 | 1.15 | 1.11 | 1.11 |

CVD, cardiovascular disease.

^a^ Relative risks were originally reported for all age groups or only part of an age group. In order to calculate relative risks for each age group, we used age-specific relative risks from meta-analyses conducted for a previous study [[3](#_ENREF_3)].

^b^ We replaced these statistically insignificant relative risks with 1 in our analysis.

^c^ We used a null association in people aged 70 years and older, because of a lack of fully convincing evidence on effects of cholesterol in older ages.

**References**

1. Danaei G, Rimm EB, Oza S, Kulkarni SC, Murray CJ, et al. (2010) The promise of prevention: the effects of four preventable risk factors on national life expectancy and life expectancy disparities by race and county in the United States. PLoS Med 7: e1000248.

2. Noda H, Iso H, Irie F, Sairenchi T, Ohtaka E, et al. (2010) Gender difference of association between LDL cholesterol concentrations and mortality from coronary heart disease amongst Japanese: the Ibaraki Prefectural Health Study. J Intern Med 267: 576-587.

3. Danaei G, Ding EL, Mozaffarian D, Taylor B, Rehm J, et al. (2009) The preventable causes of death in the United States: comparative risk assessment of dietary, lifestyle, and metabolic risk factors. PLoS Med 6: e1000058.

4. Law MR, Wald NJ, Rudnicka AR (2003) Quantifying effect of statins on low density lipoprotein cholesterol, ischaemic heart disease, and stroke: systematic review and meta-analysis. BMJ 326: 1423.

5. Lawes CM, Bennett DA, Parag V, Woodward M, Whitlock G, et al. (2003) Blood pressure indices and cardiovascular disease in the Asia Pacific region: a pooled analysis. Hypertension 42: 69-75.

6. Lawes CM, Rodgers A, Bennett DA, Parag V, Suh I, et al. (2003) Blood pressure and cardiovascular disease in the Asia Pacific region. Journal of Hypertension 21: 707-716.

7. James WPT, Jackson-Leasch R, Ni MC, Kalamara E, Shayeghi M, et al. (2004) Overweight and obesity (high body mass index). In: Ezzati M, Lopez AD, Rodgers A, Murray CJL, editors. Comparative quantification of health risks: Global and regional burden of disease attributable to selected major risk factors. Geneva: World Health Organization. pp. 497-596.

8. Parr CL, Batty GD, Lam TH, Barzi F, Fang X, et al. (2010) Body-mass index and cancer mortality in the Asia-Pacific Cohort Studies Collaboration: pooled analyses of 424,519 participants. Lancet Oncol 11: 741-752.

9. Matsuo K, Mizoue T, Tanaka K, Tsuji I, Sugawara Y, et al. Association between body-mass index (BMI) and the colorectal cancer risk in Japan: Pooled-analysis of population-based cohort studies in Japan. Annals of Oncology: In press.

10. Renehan AG, Tyson M, Egger M, Heller RF, Zwahlen M (2008) Body-mass index and incidence of cancer: a systematic review and meta-analysis of prospective observational studies. Lancet 371: 569-578.

11. Ni MC, Parag V, Nakamura M, Patel A, Rodgers A, et al. (2006) Body mass index and risk of diabetes mellitus in the Asia-Pacific region. Asia Pac J Clin Nutr 15: 127-133.
