# Supplementary material for: Adult Mortality Attributable to Preventable Risk Factors for Non-Communicable Diseases and Injuries in Japan: A Comparative Risk Assessment
Source: PLoS Med. 2012 Jan 24;9(1):e1001160. doi: 10.1371/journal.pmed.1001160 (PMC3265534; doi:10.1371/journal.pmed.1001160)
Supplement: Table S2 — Relative risks for the effects of alcohol use on disease outcomes from meta-analyses. (DOCX) [file pmed.1001160.s003.docx]

**Table S2: Relative risks for the effects of alcohol use on disease outcomes from meta-analyses.**

| **Disease outcome** | **Sex** | **Age (years)** | **I ^a^** | **II** | **III** | **IV** |
| --- | --- | --- | --- | --- | --- | --- |
| Ischemic heart disease [[1-3](#_ENREF_1)] | Both | 30–44 | 1.00 | 0.60 | 0.62 | 1.00 ^b^ |
|  |  | 45–50 | 1.00 | 0.63 | 0.65 | 1.00 ^b^ |
|  |  | 60–69 | 1.00 | 0.82 | 0.83 | 1.00 ^b^ |
|  |  | 70–79 | 1.00 | 0.92 | 0.93 | 1.00 ^b^ |
|  |  | ≥80 | 1.00 | 0.97 | 0.98 | 1.00 ^b^ |
| Ischemic stroke [[2-4](#_ENREF_2)] | Men | 30–44 | 1.00 | 0.83^b^ | 1.07 ^b^ | 3.84 |
|  |  | 45–50 | 1.00 | 0.88^b^ | 1.05 ^b^ | 2.52 |
|  |  | 60–69 | 1.00 | 0.94 ^b^ | 1.02 ^b^ | 1.69 |
|  |  | 70–79 | 1.00 | 0.97 ^b^ | 1.01 ^b^ | 1.32 |
|  |  | ≥80 | 1.00 | 1.00 ^b^ | 1.00 ^b^ | 1.00 ^b^ |
|  | Women | 30–44 | 1.00 | 0.88 ^b^ | 0.85 ^b^ | 1.33 ^b^ |
|  |  | 45–50 | 1.00 | 0.91 ^b^ | 0.89 ^b^ | 1.22 ^b^ |
|  |  | 60–69 | 1.00 | 0.96 ^b^ | 0.95 ^b^ | 1.10 ^b^ |
|  |  | 70–79 | 1.00 | 0.98 ^b^ | 0.98 ^b^ | 1.05 ^b^ |
|  |  | ≥80 | 1.00 | 1.00 ^b^ | 1.00 ^b^ | 1.00 ^b^ |
| Hemorrhagic stroke [[2-4](#_ENREF_2)] | Men | 30–44 | 1.00 | 1.65 ^b^ | 3.16 ^b^ | 6.65 |
|  |  | 45–50 | 1.00 | 1.42 ^b^ | 2.22 ^b^ | 3.60 |
|  |  | 60–69 | 1.00 | 1.19 ^b^ | 1.55 ^b^ | 2.18 |
|  |  | 70–79 | 1.00 | 1.09 ^b^ | 1.26 ^b^ | 1.55 |
|  |  | ≥80 | 1.00 | 1.00 ^b^ | 1.00 ^b^ | 1.00 ^b^ |
|  | Women | 30–44 | 1.00 | 1.30 ^b^ | 2.07 ^b^ | 3.89 ^b^ |
|  |  | 45–50 | 1.00 | 1.20 ^b^ | 1.67 ^b^ | 2.54 ^b^ |
|  |  | 60–69 | 1.00 | 1.09 ^b^ | 1.30 ^b^ | 1.70 ^b^ |
|  |  | 70–79 | 1.00 | 1.04 ^b^ | 1.14 ^b^ | 1.32 ^b^ |
|  |  | ≥80 | 1.00 | 1.00 ^b^ | 1.00 ^b^ | 1.00 ^b^ |
| Hypertensive disease [[2](#_ENREF_2),[5](#_ENREF_5)] | Men | 30≥ | 1.00 | 1.40 | 2.00 | 4.10 |
|  | Women | 30≥ | 1.00 | 1.40 | 2.00 | 2.00 |
| Cardiac arrhythmias [[2](#_ENREF_2),[6](#_ENREF_6)] | Both | 30≥ | 1.00 | 1.51 | 2.23 | 2.23 |
| Larynx cancer [[2](#_ENREF_2),[6](#_ENREF_6)] | Both | 30≥ | 1.00 | 1.83 | 3.90 | 4.93 |
| Mouth and pharynx cancer [[2](#_ENREF_2)] | Both | 30≥ | 1.00 | 1.45 | 1.85 | 5.39 |
| Selected other cancers [[3](#_ENREF_3)] | Both | 30≥ | 1.00 | 1.10 | 1.30 | 1.70 |
| Diabetes mellitus [[2](#_ENREF_2)] | Men | 30≥ | 1.00 | 0.99 | 0.57 | 0.73 |
|  | Women | 30≥ | 1.00 | 0.92 | 0.87 | 1.13 |
| Liver cirrhosis [[3](#_ENREF_3),[6](#_ENREF_6)] | Both | 30≥ | 1.00 | 1.30 | 9.50 | 13.00 |
| Pancreatitis [[2](#_ENREF_2),[5](#_ENREF_5)] | Men | 30≥ | 1.00 | 1.30 | 1.80 | 3.20 |
|  | Women | 30≥ | 1.00 | 1.30 | 1.80 | 1.80 |

I, Abstainers; II, <40 g/day of ethanol intake; III, 40–59.9 g/day; IV, ≥60 g/day.

^a^ Reference category

^b^ We replaced these statistically insignificant relative risks with 1 in our analysis.

**References**

1. Corrao G, Rubbiati L, Bagnardi V, Zambon A, Poikolainen K (2000) Alcohol and coronary heart disease: a meta-analysis. Addiction 95: 1505-1523.

2. Danaei G, Ding EL, Mozaffarian D, Taylor B, Rehm J, et al. (2009) The preventable causes of death in the United States: comparative risk assessment of dietary, lifestyle, and metabolic risk factors. PLoS Med 6: e1000058.

3. Rehm J, Room R, Monteiro M, Gmel G, Kathryn G, et al. (2004) Alcohol Use. In: Ezzati M, Lopez AD, Rodgers A, Murray CJ, editors. Comparative Quantification of health risks: Global and regional burden of disease attributable to selected major risk factors. Geneva: WHO.

4. Reynolds K, Lewis B, Nolen JD, Kinney GL, Sathya B, et al. (2003) Alcohol consumption and risk of stroke: a meta-analysis. JAMA 289: 579-588.

5. Corrao G, Bagnardi V, Zambon A, Arico S (1999) Exploring the dose-response relationship between alcohol consumption and the risk of several alcohol-related conditions: a meta-analysis. Addiction 94: 1551-1573.

6. Gutjahr E, Gmel G, Rehm J (2001) Relation between average alcohol consumption and disease: an overview. Eur Addict Res 7: 117-127.
