# Supplementary material for: Adult Mortality Attributable to Preventable Risk Factors for Non-Communicable Diseases and Injuries in Japan: A Comparative Risk Assessment
Source: PLoS Med. 2012 Jan 24;9(1):e1001160. doi: 10.1371/journal.pmed.1001160 (PMC3265534; doi:10.1371/journal.pmed.1001160)
Supplement: Table S3 — Relative risks for the effects of alcohol use on disease outcomes from Japanese studies. (DOCX) [file pmed.1001160.s004.docx]

**Table S3: Relative risks for the effects of alcohol use on disease outcomes from Japanese studies.**

| **Disease outcome** | **Men** |  | **Women** |  |
| --- | --- | --- | --- | --- |
| Breast cancer [[1](#_ENREF_1)] |  |  | Never drinker ^d^ | 1.00 |
|  |  |  | Ex-drinker | 1.41 |
|  |  |  | Occasional drinker | 1.17 ^e^ |
|  |  |  | 1–2 times/week | 1.25 ^e^ |
|  |  |  | 3–4 times/week | 0.78 ^e^ |
|  |  |  | ≥5–8 times/week | 1.56 |
| Colorectal cancer [[2](#_ENREF_2)] | Never/ex-drinker ^d^ | 1.00 | Never/ex-drinker ^d^ | 1.00 |
|  | Occasional drinkers | 1.00 ^e^ | Occasional drinkers | 0.96 ^e^ |
|  | <23.0 g/day | 1.22 ^e^ | <23.0 g/day | 0.93 ^e^ |
|  | 23.0–45.9 g/day | 1.42 | ≥23.0 g/day | 1.57 |
|  | 46.0–68.9 g/day | 1.95 |  |  |
|  | 69.0–91.9 g/day | 2.15 |  |  |
|  | ≥92.0 g/day | 2.96 |  |  |
| Esophagus cancer [[3](#_ENREF_3)]^a^ | Non-drinker ^d^ | 1.00 | Non-drinker ^d^ | 1.00 |
|  | Occasional drinkers | 0.60 ^e^ | Occasional drinkers | 0.60 ^e^ |
|  | <150.0 g/week | 1.64 ^e^ | <150.0 g/week | 1.64 ^e^ |
|  | 150.0–299.9 g/week | 2.59 | 150.0–299.9 g/week | 2.59 |
|  | ≥300.0 g/week | 4.64 | ≥300.0 g/week | 4.64 |
| Liver cancer [[4](#_ENREF_4)] | Never/ex-drinker | 1.70 | Never/ex-drinker | 1.50 ^e^ |
|  | Occasional drinker ^d^ | 1.00 | Occasional drinker ^d^ | 1.00 |
|  | <23.0 g/day | 0.88 ^e^ | <23.0 g/day | 0.86 ^e^ |
|  | 23.0–45.9 g/day | 1.06 ^e^ | ≥23.0 g/day | 3.60 |
|  | 46.0–68.9 g/day | 1.07 ^e^ |  |  |
|  | 69.0–91.9 g/day | 1.76 |  |  |
|  | ≥92.0 g/day | 1.66 ^e^ |  |  |
| Road traffic injury [[5](#_ENREF_5)]^b^ | BAC<0.25 mg/L ^d^ | 1.00 | BAC<0.25 mg/L ^d^ | 1.00 |
|  | ≥0.25 mg/L | 8.00 | ≥0.25 mg/L | 8.00 |
| Suicide [[6](#_ENREF_6)] ^a, c^ | Never/ex-drinker | 1.80 ^e^ | Never/ex-drinker | 1.80 ^e^ |
|  | Occasional drinker ^d^ | 1.00 | Occasional drinker ^d^ | 1.00 |
|  | <138.0 g/week | 1.20 ^e^ | <138.0 g/week | 1.20 ^e^ |
|  | 138.0–251.9 g/week | 1.40 ^e^ | 138.0–251.9 g/week | 1.40 ^e^ |
|  | 252.0–413.9 g/week | 1.30 ^e^ | 252.0–413.9 g/week | 1.30 ^e^ |
|  | ≥414 g/week | 2.10 | ≥414 g/week | 2.10 |

BAC, Breath alcohol concentration.

^a^ Relative risks were reported for males only. We applied same values to females.

^b^ Relative risks were reported for both sexes combined.

^c^ Relative risks were estimated for suicide, and we applied same values to falls, homicide, and other injuries. These estimates excluded the first 2 years of follow-up.

^d^ Reference category

^e^ We replaced these statistically insignificant relative risks with 1 in our analysis.

**References**

1. Suzuki R, Iwasaki M, Inoue M, Sasazuki S, Sawada N, et al. (2010) Alcohol consumption-associated breast cancer incidence and potential effect modifiers: the Japan Public Health Center-based Prospective Study. Int J Cancer 127: 685-695.

2. Mizoue T, Inoue M, Wakai K, Nagata C, Shimazu T, et al. (2008) Alcohol drinking and colorectal cancer in Japanese: a pooled analysis of results from five cohort studies. Am J Epidemiol 167: 1397-1406.

3. Ishiguro S, Sasazuki S, Inoue M, Kurahashi N, Iwasaki M, et al. (2009) Effect of alcohol consumption, cigarette smoking and flushing response on esophageal cancer risk: a population-based cohort study (JPHC study). Cancer Lett 275: 240-246.

4. Shimazu T, Sasazuki S, Wakai K, Tamakoshi A, Tsuji I, et al. (In press) Alcohol drinking and primary liver cancer: A pooled analysis of four Japanese cohort studies. International Journal of Cancer.

5. Hitosugi M, Sorimachi Y, Kurosu A, Nagai T, Tokudome S (2003) Risk of death due to alcohol-impaired driving in Japan. Lancet 361: 1132.

6. Akechi T, Iwasaki M, Uchitomi Y, Tsugane S (2006) Alcohol consumption and suicide among middle-aged men in Japan. Br J Psychiatry 188: 231-236.
