# Supplementary material for: Adult Mortality Attributable to Preventable Risk Factors for Non-Communicable Diseases and Injuries in Japan: A Comparative Risk Assessment
Source: PLoS Med. 2012 Jan 24;9(1):e1001160. doi: 10.1371/journal.pmed.1001160 (PMC3265534; doi:10.1371/journal.pmed.1001160)
Supplement: Table S4 — Relative risks for the effects of tobacco smoking on disease outcomes. (DOCX) [file pmed.1001160.s005.docx]

**Table S4: Relative risks for the effects of tobacco smoking on disease outcomes.**

| **Disease outcome** ^a^ | **Sex** | **Age (years)** | | | | |
| --- | --- | --- | --- | --- | --- | --- |
|  |  | **30–44** | **45–59** | **60–69** | **70–79** | **≥80** |
| Ischemic heart disease [[1](#_ENREF_1)] ^b^ | Men | 4.08 | 2.50 | 2.19 | 1.92 | 1.09 ^c^ |
|  | Women | 2.47 | 4.36 | 3.10 | 2.21 | 1.64 |
| Total stroke [[1](#_ENREF_1)] ^b^ | Men | 1.41 | 1.41 | 1.26 | 1.13 ^c^ | 1.02 ^c^ |
|  | Women | 2.75 | 2.75 | 1.85 | 1.24 ^c^ | 0.98 ^c^ |
| Aortic aneurysms and dissection [[2](#_ENREF_2)] ^b^ | Men | 8.04 | 3.89 | 3.89 | 3.89 | 1.20 ^c^ |
|  | Women | 1.69 ^c^ | 2.35 | 2.35 | 2.35 | 1.70 |
| Bladder cancer [[2](#_ENREF_2)] | Men | 5.35 | 5.35 | 5.35 | 5.35 | 5.35 |
|  | Women | 1.86 ^c^ | 1.86 ^c^ | 1.86 ^c^ | 1.86 ^c^ | 1.86 ^c^ |
| Cervix uteri cancer [[2](#_ENREF_2)] | Women | 2.32 | 2.32 | 2.32 | 2.32 | 2.32 |
| Esophagus cancer [[2](#_ENREF_2)] | Men | 3.39 | 3.39 | 3.39 | 3.39 | 3.39 |
|  | Women | 1.90 ^c^ | 1.90 ^c^ | 1.90 ^c^ | 1.90 ^c^ | 1.90c |
| Kidney cancer [[2](#_ENREF_2)] | Men | 1.57 ^c^ | 1.57 ^c^ | 1.57 ^c^ | 1.57 ^c^ | 1.57 ^c^ |
|  | Women | 0.60 ^c^ | 0.60 ^c^ | 0.60 ^c^ | 0.60 ^c^ | 0.60 ^c^ |
| Leukemia [[2](#_ENREF_2)] | Men | 1.45 ^c^ | 1.45 ^c^ | 1.45 ^c^ | 1.45 ^c^ | 1.45 ^c^ |
|  | Women | 0.96 ^c^ | 0.96 ^c^ | 0.96 ^c^ | 0.96 ^c^ | 0.96 ^c^ |
| Liver cancer [[2](#_ENREF_2)] | Men | 1.81 | 1.81 | 1.81 | 1.81 | 1.81 |
|  | Women | 1.73 | 1.73 | 1.73 | 1.73 | 1.73 |
| Lung cancer [[2](#_ENREF_2)] | Men | 4.79 | 4.79 | 4.79 | 4.79 | 4.79 |
|  | Women | 3.88 | 3.88 | 3.88 | 3.88 | 3.88 |
| Mouth and pharynx cancer [[2](#_ENREF_2)] | Men | 2.66 | 2.66 | 2.66 | 2.66 | 2.66 |
|  | Women | 1.97 ^c^ | 1.97 ^c^ | 1.97 ^c^ | 1.97 ^c^ | 1.97 ^c^ |
| Pancreatic cancer [[2](#_ENREF_2)] | Men | 1.58 | 1.58 | 1.58 | 1.58 | 1.58 |
|  | Women | 1.81 | 1.81 | 1.81 | 1.81 | 1.81 |
| Stomach cancer [[2](#_ENREF_2)] | Men | 1.51 | 1.51 | 1.51 | 1.51 | 1.51 |
|  | Women | 1.22 ^c^ | 1.22 ^c^ | 1.22 ^c^ | 1.22 ^c^ | 1.22 ^c^ |
| Chronic obstructive pulmonary disease [[2](#_ENREF_2)] | Men | 3.09 | 3.09 | 3.09 | 3.09 | 3.09 |
|  | Women | 3.55 | 3.55 | 3.55 | 3.55 | 3.55 |
| Asthma [[3](#_ENREF_3)] | Men | 1.25 ^c^ | 1.25 ^c^ | 1.25 ^c^ | 1.25 ^c^ | 1.25 ^c^ |
|  | Women | 3.46 | 3.46 | 3.46 | 3.46 | 3.46 |
| Lower respiratory tract infection [[2](#_ENREF_2)] | Men | 1.17 ^c^ | 1.17 ^c^ | 1.17 ^c^ | 1.17 ^c^ | 1.17 ^c^ |
|  | Women | 1.39 | 1.39 | 1.39 | 1.39 | 1.39 |

^a^ Relative risks for diabetes mellitus and tuberculosis were not reported from the pooled cohort study.

^b^ Relative risks were originally reported only for people aged 40–79 years old. In order to calculate relative risks for each age group, we used age-specific relative risks from meta-analyses conducted for a previous study [[4](#_ENREF_4)].

^c^ We replaced these statistically insignificant relative risks with 1 in our analysis.

**References**

1. Honjo K, Iso H, Tsugane S, Tamakoshi A, Satoh H, et al. (2010) The effects of smoking and smoking cessation on mortality from cardiovascular disease among Japanese: pooled analysis of three large-scale cohort studies in Japan. Tob Control 19: 50-57.

2. Katanoda K, Marugame T, Saika K, Satoh H, Tajima K, et al. (2008) Population attributable fraction of mortality associated with tobacco smoking in Japan: a pooled analysis of three large-scale cohort studies. J Epidemiol 18: 251-264.

3. Sobue T (2008) Research on collection of scientific evidence on tobacco smoking (in Japanese). Report to the Ministry of Health, Labour and Welfare for 2005-2007 Grants-in-Aid for Scientific Research. Tokyo: National Cancer Center.

4. Danaei G, Ding EL, Mozaffarian D, Taylor B, Rehm J, et al. (2009) The preventable causes of death in the United States: comparative risk assessment of dietary, lifestyle, and metabolic risk factors. PLoS Med 6: e1000058.
