# Supplementary material for: Adult Mortality Attributable to Preventable Risk Factors for Non-Communicable Diseases and Injuries in Japan: A Comparative Risk Assessment
Source: PLoS Med. 2012 Jan 24;9(1):e1001160. doi: 10.1371/journal.pmed.1001160 (PMC3265534; doi:10.1371/journal.pmed.1001160)
Supplement: Table S5 — Relative risks for the effects of physical inactivity on disease outcomes. (DOCX) [file pmed.1001160.s006.docx]

**Table S5: Relative risks for the effects of physical inactivity on disease outcomes.**

| **Disease outcome ^a^** | **Age (years) ^b^** | **I ^c,d^** | **II** | **III** |
| --- | --- | --- | --- | --- |
| Ischemic heart disease | 30–69 | 1.00 | 1.44 | 1.71 |
|  | 70–79 | 1.00 | 1.31 | 1.50 |
|  | ≥80 | 1.00 | 1.20 | 1.30 |
| Ischemic stroke | 30–69 | 1.00 | 1.10^e^ | 1.53 |
|  | 70–79 | 1.00 | 1.08 ^e^ | 1.38 |
|  | ≥80 | 1.00 | 1.05 ^e^ | 1.24 |
| Breast cancer | 30–44 | 1.00 | 1.13 | 1.25 |
|  | 45–69 | 1.00 | 1.13 | 1.34 |
|  | 70–79 | 1.00 | 1.09 | 1.25 |
|  | ≥80 | 1.00 | 1.06 ^e^ | 1.16 |
| Colon cancer | 30–69 | 1.00 | 1.18 | 1.68 |
|  | 70–79 | 1.00 | 1.13 | 1.48 |
|  | ≥80 | 1.00 | 1.08 ^e^ | 1.30 |
| Diabetes mellitus | 30–69 | 1.00 | 1.24 | 1.45 |
|  | 70–79 | 1.00 | 1.18 | 1.32 |
|  | ≥80 | 1.00 | 1.11 ^e^ | 1.20 |

^a^ We obtained all relative risks from Bull et al. (2004) [[1](#_ENREF_1)].

^b^ Except for breast cancer, relative risks were estimated for both sexes combined.

^c^ Bull et al. (2004) originally defined the categories as: I, “≥2.5 hr/wk of moderate activity or ≥1 hr/wk of vigorous activity”; II, “<2.5h/wk of moderate activity or < 1h/wk of vigorous activity”; III, “no moderate or vigorous activity”. We used self-reports on the intensity of physical activity and substituted “highly intense”, “moderately intense”, and “poorly intense” for I, II, and III, respectively.

^d^ Reference category

^e^ We replaced these statistically insignificant relative risks with 1 in our analysis.

**References**

1. Bull FC, Armstrong TP, Dixon T, Ham S, Neiman A, et al. (2004) Physical inactivity. In: Ezzati M, Lopez AD, Rodgers A, Murray CJL, editors. Comparative quantification of health risks: global and regional burden of disease attributable to selected major risk factors. Geneva: World Health Organization. pp. 729-881.
