# Supplementary material for: Adult Mortality Attributable to Preventable Risk Factors for Non-Communicable Diseases and Injuries in Japan: A Comparative Risk Assessment
Source: PLoS Med. 2012 Jan 24;9(1):e1001160. doi: 10.1371/journal.pmed.1001160 (PMC3265534; doi:10.1371/journal.pmed.1001160)
Supplement: Table S6 — Relative risks for the effects of dietary risk factors on disease outcomes. (DOCX) [file pmed.1001160.s007.docx]

**Table S6: Relative risks for the effects of dietary risk factors on disease outcomes.**

| **Risk factor, disease outcome** | **Unit** | **Age (years)** | | | | | |
| --- | --- | --- | --- | --- | --- | --- | --- |
|  |  | **30-44** | **45-59** | **60-69** | **70-79** | **≥80** | **≥30** |
| *High TFA intake* |  |  |  |  |  |  |  |
| Ischemic heart disease [[1](#_ENREF_1)] | Per 1 percentage point more calories | 1.40 | 1.29 | 1.14 | 1.08 | 1.06 |  |
| *Low PUFA intake* |  |  |  |  |  |  |  |
| Ischemic heart disease [[2](#_ENREF_2)] ^a^ | Per 1 percentage point less calories | 1.14 | 1.10 | 1.05 | 1.03 | 1.02 |  |
| *High dietary sodium intake* |  |  |  |  |  |  |  |
| SBP [[3](#_ENREF_3)] | mmHg SBP per 100 mmol/d increase |  |  |  |  |  | 7.11 (SBP≥140)  3.27 (SBP<140) |
| Stomach cancer [[3](#_ENREF_3),[4](#_ENREF_4)] | Per 100 mmol/d increase |  |  |  |  |  | 1.57 |
| *Low fruit and vegetable intake* |  | 1.04 | 1.04 | 1.04 | 1.03 ^d^ | 1.02 ^d^ |  |
| Ischemic heart disease [[3](#_ENREF_3),[5](#_ENREF_5)] | Per 80 g/d decrease | 1.06 | 1.06 | 1.06 | 1.05 | 1.03 ^d^ |  |
| Ischemic stroke [[3](#_ENREF_3),[6](#_ENREF_6)] | Per 80 g/d decrease | 1.01^d^ | 1.01 ^d^ | 1.01 ^d^ | 1.01 ^d^ | 1.00 ^d^ |  |
| Colorectal cancer [[3](#_ENREF_3),[7](#_ENREF_7)] | Per 80 g/d decrease | 1.10 | 1.10 | 1.10 | 1.08 ^d^ | 1.05 ^d^ |  |
| Esophagus cancer [[8](#_ENREF_8)]^a, b, c^ | Per 100 g/d decrease | 1.04 | 1.04 | 1.04 | 1.03 ^d^ | 1.02 ^d^ |  |
| Lung cancer [[3](#_ENREF_3),[7](#_ENREF_7)] | Per 80 g/d decrease | 1.40 | 1.29 | 1.14 | 1.08 | 1.06 |  |
| Stomach cancer [[3](#_ENREF_3),[7](#_ENREF_7)] | Per 80 g/d decrease | 1.06 ^d^ | 1.06 ^d^ | 1.06 ^d^ | 1.05 ^d^ | 1.03 ^d^ |  |

SBP, systolic blood pressure.

^a^ Relative risks were reported for all ages or the specified age group. We used the age gradients of the relative risks from meta-analyses conducted for the US study to calculate relative risks for each age group [[3](#_ENREF_3)].

^b^ Relative risks were estimated for males only, and we applied the same values to females.

^c^ We applied these relative risks for esophagus cancers to mouth and pharyngeal cancers.

^d^ We replaced these statistically insignificant relative risks with 1 in our analysis.

**References**

1. Mozaffarian D, Katan MB, Ascherio A, Stampfer MJ, Willett WC (2006) Trans fatty acids and cardiovascular disease. N Engl J Med 354: 1601-1613.

2. Mozaffarian D, Micha R, Wallace S (2010) Effects on coronary heart disease of increasing polyunsaturated fat in place of saturated fat: a systematic review and meta-analysis of randomized controlled trials. PLoS Med 7: e1000252.

3. Danaei G, Ding EL, Mozaffarian D, Taylor B, Rehm J, et al. (2009) The preventable causes of death in the United States: comparative risk assessment of dietary, lifestyle, and metabolic risk factors. PLoS Med 6: e1000058.

4. World Cancer Research Fund, American Institute for Cancer Research (2007) Food, nutrition, physical activity, and prevention of cancer: a global perspective. Washington, D.C.: American Institute for Cancer Research.

5. Dauchet L, Amouyel P, Hercberg S, Dallongeville J (2006) Fruit and vegetable consumption and risk of coronary heart disease: a meta-analysis of cohort studies. J Nutr 136: 2588-2593.

6. Dauchet L, Amouyel P, Dallongeville J (2005) Fruit and vegetable consumption and risk of stroke: a meta-analysis of cohort studies. Neurology 65: 1193-1197.

7. Lock K, Pomerleau J, Causer L, McKee M (2004) Low fruit and vegetable consumption. In: Ezzati M, Lopez AD, Rodgers A, Murray CJ, editors. Comparative quantification of health risks: global and regional burden of disease attributable to selected major risk factors. Geneva: World Health Organization. pp. 597-728.

8. Yamaji T, Inoue M, Sasazuki S, Iwasaki M, Kurahashi N, et al. (2008) Fruit and vegetable consumption and squamous cell carcinoma of the esophagus in Japan: the JPHC study. Int J Cancer 123: 1935-1940.
