# Supplementary material for: Adult Mortality Attributable to Preventable Risk Factors for Non-Communicable Diseases and Injuries in Japan: A Comparative Risk Assessment
Source: PLoS Med. 2012 Jan 24;9(1):e1001160. doi: 10.1371/journal.pmed.1001160 (PMC3265534; doi:10.1371/journal.pmed.1001160)
Supplement: Table S7 — Relative risks for the effects of infections on disease outcomes. (DOCX) [file pmed.1001160.s008.docx]

**Table S7: Relative risks for the effects of infections on disease outcomes.**

| **Agent, disease outcome** | **Units** |  |
| --- | --- | --- |
| *Hepatitis B virus* |  |  |
| Liver cancer [[1](#_ENREF_1)] | Anti-HCV (-) & HBsAg (-) ^b^ | 1 |
|  | Anti-HCV (-) & HBsAg (+) | 74 |
| *Hepatitis C virus* |  |  |
| Liver cancer [[1](#_ENREF_1)] | Anti-HCV (-) & HBsAg (-) ^b^ | 1 |
|  | Anti-HCV (+) & HBsAg (-) | 36 |
| *Helicobacter pylori* |  |  |
| Stomach cancer ^a^ | Anti-*Helicobacter pylori* immunoglobulin G  antibody sero-positivity | 2.9 |

HCV, hepatitis C virus; HBsAg, hepatitis B surface antigen.

^a^ We conducted an ad-hoc analysis for this study.

^b^ Reference category

**References**

1. Tanaka H, Tsukuma H, Yamano H, Oshima A, Shibata H (2004) Prospective study on the risk of hepatocellular carcinoma among hepatitis C virus-positive blood donors focusing on demographic factors, alanine aminotransferase level at donation and interaction with hepatitis B virus. International Journal of Cancer 112: 1075-1080.
