# Supplementary material for: Adult Mortality Attributable to Preventable Risk Factors for Non-Communicable Diseases and Injuries in Japan: A Comparative Risk Assessment
Source: PLoS Med. 2012 Jan 24;9(1):e1001160. doi: 10.1371/journal.pmed.1001160 (PMC3265534; doi:10.1371/journal.pmed.1001160)
Supplement: Table S9 — Population-attributable fractions of cause-specific mortality attributable to individual risk factors in women in 2007. (DOCX) [file pmed.1001160.s010.docx]

**Table S9: Population-attributable fractions of cause-specific mortality attributable to individual risk factors in women in 2007.**

| **Risk factor, disease** | **Age (years)** | | | | | | | | | | | |
| --- | --- | --- | --- | --- | --- | --- | --- | --- | --- | --- | --- | --- |
|  | **20–29** | | **30–44** | | **45–59** | | **60–69** | | **70–79** | | **≥80** | |
| *High blood glucose* |  |  |  |  |  |  |  |  |  |  |  |  |
| Ischemic heart disease |  |  | 0.13 | (0.00, 0.27) | 0.22 | (0.13, 0.31) | 0.14 | (0.04, 0.21) | 0.14 | (0.06, 0.22) | 0.14 | (0.06, 0.23) |
| Total stroke |  |  | 0.12 | (0.01, 0.24) | 0.19 | (0.01, 0.37) | 0.18 | (0.10, 0.26) | 0.00 | (0.00, 0.00) | 0.00 | (0.00, 0.00) |
| Diabetes mellitus |  |  | 1.00 |  | 1.00 |  | 1.00 |  | 1.00 |  | 1.00 |  |
| *High LDL cholesterol* |  |  |  |  |  |  |  |  |  |  |  |  |
| Ischemic heart disease |  |  | 0.00 | (0.00, 0.00) | 0.35 | (0.00, 0.57) | 0.22 | (0.09, 0.33) | 0.19 | (0.06, 0.29) | 0.12 | (0.04, 0.21) |
| Ischemic stroke |  |  | 0.19 | (0.00, 0.34) | 0.30 | (0.12, 0.46) | 0.21 | (0.10, 0.30) | 0.00 | (0.00, 0.00) | 0.00 | (0.00, 0.00) |
| *High blood pressure* |  |  |  |  |  |  |  |  |  |  |  |  |
| Ischemic heart disease |  |  | 0.00 | (0.00, 0.00) | 0.19 | (0.12, 0.25) | 0.32 | (0.24, 0.38) | 0.18 | (0.03, 0.31) | 0.20 | (0.02, 0.34) |
| Total stroke |  |  | 0.00 | (0.00, 0.00) | 0.24 | (0.16, 0.32) | 0.32 | (0.22, 0.40) | 0.24 | (0.15, 0.34) | 0.28 | (0.16, 0.40) |
| Hypertensive disease |  |  | 0.00 | (0.00, 0.00) | 0.67 | (0.51, 0.81) | 0.85 | (0.74, 0.93) | 0.78 | (0.66, 0.87) | 0.75 | (0.66, 0.84) |
| Other selected CVD |  |  | 0.00 | (0.00, 0.00) | 0.42 | (0.30, 0.55) | 0.61 | (0.48, 0.73) | 0.52 | (0.42, 0.64) | 0.49 | (0.41, 0.58) |
| *High body mass index* |  |  |  |  |  |  |  |  |  |  |  |  |
| Ischemic heart disease |  |  | 0.00 | (0.00, 0.00) | 0.09 | (0.06, 0.12) | 0.12 | (0.08, 0.18) | 0.09 | (0.04, 0.14) | 0.03 | (0.00, 0.05) |
| Ischemic stroke |  |  | 0.00 | (0.00, 0.00) | 0.10 | (0.05, 0.15) | 0.12 | (0.06, 0.18) | 0.09 | (0.04, 0.15) | 0.00 | (0.00, 0.00) |
| Hypertensive disease |  |  | 0.00 | (0.00, 0.00) | 0.00 | (0.00, 0.00) | 0.21 | (0.06, 0.34) | 0.18 | (0.05, 0.30) | 0.00 | (0.00, 0.00) |
| Postmenopausal breast cancer |  |  |  |  | 0.04 | (0.01, 0.07) | 0.06 | (0.00, 0.11) | 0.06 | (0.01, 0.11) | 0.04 | (0.01, 0.08) |
| Colon cancer |  |  | 0.00 | (0.00, 0.00) | 0.03 | (0.01, 0.06) | 0.05 | (0.02, 0.09) | 0.05 | (0.02, 0.09) | 0.04 | (0.01, 0.07) |
| Corpus uteri cancer |  |  | 0.00 | (0.00, 0.00) | 0.10 | (0.08, 0.12) | 0.15 | (0.12, 0.19) | 0.16 | (0.13, 0.19) | 0.11 | (0.07, 0.16) |
| Kidney cancer |  |  | 0.00 | (0.00, 0.00) | 0.06 | (0.05, 0.08) | 0.10 | (0.07, 0.13) | 0.10 | (0.08, 0.13) | 0.07 | (0.04, 0.11) |
| Pancreatic cancer |  |  | 0.00 | (0.00, 0.00) | 0.03 | (0.00, 0.05) | 0.04 | (0.01, 0.07) | 0.04 | (0.01, 0.07) | 0.03 | (0.01, 0.05) |
| Diabetes mellitus |  |  | 0.00 | (0.00, 0.00) | 0.19 | (0.14, 0.24) | 0.23 | (0.16, 0.30) | 0.18 | (0.10, 0.25) | 0.12 | (0.07, 0.19) |
| *Alcohol use* |  |  |  |  |  |  |  |  |  |  |  |  |
| Ischemic heart disease |  |  | -0.16 | (-0.22, -0.10) | -0.14 | (-0.19, -0.08) | -0.04 | (-0.06, -0.02) | -0.01 | (-0.02, -0.01) | 0.00 | (0.00, 0.00) |
| Ischemic stroke |  |  | 0.00 | (0.00, 0.00) | 0.00 | (0.00, 0.00) | 0.00 | (0.00, 0.00) | 0.00 | (0.00, 0.00) | 0.00 | (0.00, 0.00) |
| Hemorrhagic stroke |  |  | 0.00 | (0.00, 0.00) | 0.00 | (0.00, 0.00) | 0.00 | (0.00, 0.00) | 0.00 | (0.00, 0.00) | 0.00 | (0.00, 0.00) |
| Hypertensive disease |  |  | 0.25 | (0.22, 0.29) | 0.20 | (0.17, 0.23) | 0.13 | (0.11, 0.16) | 0.08 | (0.06, 0.10) | 0.05 | (0.03, 0.07) |
| Cardiac arrhythmias |  |  | 0.30 | (0.26, 0.33) | 0.24 | (0.21, 0.28) | 0.16 | (0.13, 0.19) | 0.09 | (0.07, 0.12) | 0.06 | (0.04, 0.08) |
| Breast cancer |  |  | 0.08 | (0.02, 0.16) | 0.08 | (0.01, 0.15) | 0.06 | (0.01, 0.12) | 0.03 | (0.01, 0.06) | 0.04 | (0.01, 0.09) |
| Colon cancer |  |  | 0.10 | (0.02, 0.18) | 0.07 | (0.01, 0.14) | 0.04 | (0.01, 0.08) | 0.01 | (0.00, 0.02) | 0.01 | (0.00, 0.02) |
| Esophagus cancer |  |  | 0.31 | (0.21, 0.42) | 0.23 | (0.15, 0.34) | 0.13 | (0.07, 0.20) | 0.04 | (0.01, 0.08) | 0.02 | (0.00, 0.05) |
| Larynx cancer |  |  | 0.50 | (0.44, 0.57) | 0.42 | (0.36, 0.48) | 0.30 | (0.25, 0.35) | 0.17 | (0.13, 0.21) | 0.10 | (0.06, 0.14) |
| Liver cancer |  |  | 0.33 | (0.05, 0.66) | 0.26 | (0.03, 0.57) | 0.15 | (0.01, 0.39) | 0.04 | (0.00, 0.15) | 0.02 | (0.00, 0.11) |
| Mouth cancer |  |  | 0.39 | (0.32, 0.47) | 0.30 | (0.24, 0.37) | 0.19 | (0.15, 0.24) | 0.09 | (0.07, 0.13) | 0.05 | (0.03, 0.07) |
| Pharynx cancer |  |  | 0.39 | (0.32, 0.47) | 0.30 | (0.24, 0.37) | 0.19 | (0.15, 0.25) | 0.09 | (0.07, 0.13) | 0.05 | (0.03, 0.07) |
| Other selected cancers |  |  | 0.12 | (0.10, 0.14) | 0.08 | (0.07, 0.10) | 0.05 | (0.04, 0.06) | 0.02 | (0.02, 0.03) | 0.01 | (0.01, 0.02) |
| Diabetes mellitus |  |  | -0.03 | (-0.03, -0.02) | -0.03 | (-0.03, -0.02) | -0.02 | (-0.02, -0.01) | -0.01 | (-0.02, -0.01) | -0.01 | (-0.01, -0.01) |
| Liver cirrhosis |  |  | 0.72 | (0.64, 0.80) | 0.62 | (0.54, 0.71) | 0.48 | (0.38, 0.58) | 0.23 | (0.15, 0.33) | 0.10 | (0.03, 0.20) |
| Pancreatitis |  |  | 0.21 | (0.14, 0.29) | 0.17 | (0.11, 0.22) | 0.11 | (0.07, 0.15) | 0.06 | (0.04, 0.09) | 0.04 | (0.02, 0.06) |
| Falls | 0.02 | (0.00, 0.06) | 0.03 | (0.00, 0.08) | 0.01 | (0.00, 0.05) | 0.00 | (0.00, 0.02) | 0.00 | (0.00, 0.00) | 0.00 | (0.00, 0.00) |
| Homicide | 0.02 | (0.00, 0.06) | 0.03 | (0.00, 0.08) | 0.01 | (0.00, 0.04) | 0.00 | (0.00, 0.02) | 0.00 | (0.00, 0.00) | 0.00 | (0.00, 0.00) |
| Road traffic accident | 0.01 | (0.00, 0.00) | 0.01 | (0.00, 0.00) | 0.01 | (0.00, 0.00) | 0.01 | (0.00, 0.00) | 0.01 | (0.00, 0.00) | 0.01 | (0.00, 0.00) |
| Suicide | 0.02 | (0.00, 0.06) | 0.03 | (0.00, 0.08) | 0.01 | (0.00, 0.05) | 0.00 | (0.00, 0.02) | 0.00 | (0.00, 0.00) | 0.00 | (0.00, 0.00) |
| *Tobacco smoking* |  |  |  |  |  |  |  |  |  |  |  |  |
| Ischemic heart disease |  |  | 0.00 | (0.00, 0.00) | 0.27 | (0.09, 0.49) | 0.33 | (0.14, 0.66) | 0.19 | (0.09, 0.34) | 0.13 | (0.02, 0.32) |
| Total stroke |  |  | 0.00 | (0.00, 0.87) | 0.16 | (0.05, 0.33) | 0.13 | (0.05, 0.36) | 0.00 | (0.00, 0.00) | 0.00 | (0.00, 0.00) |
| Aortic aneurysms and dissection |  |  | 0.00 | (0.00, 0.00) | 0.13 | (0.01, 0.38) | 0.24 | (0.06, 0.57) | 0.21 | (0.02, 0.48) | 0.14 | (0.01, 0.44) |
| Bladder cancer |  |  | 0.00 | (0.00, 0.00) | 0.00 | (0.00, 0.00) | 0.00 | (0.00, 0.00) | 0.00 | (0.00, 0.00) | 0.00 | (0.00, 0.00) |
| Cervix uteri cancer |  |  | 0.00 | (0.00, 0.86) | 0.13 | (0.02, 0.31) | 0.24 | (0.05, 0.59) | 0.20 | (0.05, 0.43) | 0.23 | (0.06, 0.49) |
| Esophagus cancer |  |  | 0.00 | (0.00, 0.00) | 0.00 | (0.00, 0.00) | 0.00 | (0.00, 0.00) | 0.00 | (0.00, 0.00) | 0.00 | (0.00, 0.00) |
| Kidney cancer |  |  | 0.00 | (0.00, 0.00) | 0.00 | (0.00, 0.00) | 0.00 | (0.00, 0.00) | 0.00 | (0.00, 0.00) | 0.00 | (0.00, 0.00) |
| Leukemia |  |  | 0.00 | (0.00, 0.00) | 0.00 | (0.00, 0.00) | 0.00 | (0.00, 0.00) | 0.00 | (0.00, 0.00) | 0.00 | (0.00, 0.00) |
| Liver cancer |  |  | 0.00 | (0.00, 0.76) | 0.07 | (0.01, 0.20) | 0.15 | (0.03, 0.41) | 0.12 | (0.04, 0.27) | 0.14 | (0.04, 0.32) |
| Lung cancer |  |  | 0.00 | (0.00, 0.92) | 0.24 | (0.09, 0.44) | 0.40 | (0.20, 0.70) | 0.36 | (0.24, 0.52) | 0.39 | (0.25, 0.62) |
| Mouth cancer |  |  | 0.00 | (0.00, 0.00) | 0.00 | (0.00, 0.00) | 0.00 | (0.00, 0.00) | 0.00 | (0.00, 0.00) | 0.00 | (0.00, 0.00) |
| Pancreatic cancer |  |  | 0.00 | (0.00, 0.78) | 0.08 | (0.02, 0.22) | 0.16 | (0.04, 0.45) | 0.13 | (0.04, 0.27) | 0.15 | (0.06, 0.36) |
| Pharynx cancer |  |  | 0.00 | (0.00, 0.00) | 0.00 | (0.00, 0.00) | 0.00 | (0.00, 0.00) | 0.00 | (0.00, 0.00) | 0.00 | (0.00, 0.00) |
| Stomach cancer |  |  | 0.00 | (0.00, 0.00) | 0.00 | (0.00, 0.00) | 0.00 | (0.00, 0.00) | 0.00 | (0.00, 0.00) | 0.00 | (0.00, 0.00) |
| Chronic obstructive pulmonary disease |  |  | 0.00 | (0.00, 0.92) | 0.22 | (0.04, 0.53) | 0.37 | (0.08, 0.76) | 0.33 | (0.09, 0.64) | 0.36 | (0.10, 0.70) |
| Asthma |  |  | 0.00 | (0.00, 0.93) | 0.21 | (0.05, 0.51) | 0.36 | (0.12, 0.70) | 0.32 | (0.13, 0.58) | 0.36 | (0.13, 0.65) |
| Lower respiratory tract infection |  |  | 0.00 | (0.00, 0.65) | 0.04 | (0.00, 0.14) | 0.08 | (0.00, 0.28) | 0.07 | (0.00, 0.17) | 0.08 | (0.00, 0.22) |
| *Physical inactivity* |  |  |  |  |  |  |  |  |  |  |  |  |
| Ischemic heart disease |  |  | 0.27 | (0.24, 0.31) | 0.26 | (0.22, 0.29) | 0.27 | (0.23, 0.31) | 0.18 | (0.14, 0.22) | 0.18 | (0.13, 0.22) |
| Ischemic stroke |  |  | 0.16 | (0.10, 0.22) | 0.15 | (0.09, 0.20) | 0.16 | (0.09, 0.22) | 0.08 | (0.04, 0.13) | 0.12 | (0.04, 0.20) |
| Breast cancer |  |  | 0.11 | (0.08, 0.14) | 0.13 | (0.10, 0.15) | 0.13 | (0.11, 0.16) | 0.08 | (0.06, 0.11) | 0.08 | (0.06, 0.11) |
| Colon cancer |  |  | 0.23 | (0.19, 0.26) | 0.21 | (0.17, 0.25) | 0.22 | (0.18, 0.26) | 0.14 | (0.10, 0.17) | 0.15 | (0.10, 0.19) |
| Diabetes mellitus |  |  | 0.19 | (0.15, 0.22) | 0.17 | (0.14, 0.21) | 0.18 | (0.15, 0.22) | 0.12 | (0.08, 0.16) | 0.10 | (0.07, 0.14) |
| *High TFA intake* |  |  |  |  |  |  |  |  |  |  |  |  |
| Ischemic heart disease |  |  | 0.00 | (0.00, 0.00) | 0.00 | (0.00, 0.00) | 0.00 | (0.00, 0.00) | 0.00 | (0.00, 0.00) | 0.00 | (0.00, 0.00) |
| *Low PUFA intake* |  |  |  |  |  |  |  |  |  |  |  |  |
| Ischemic heart disease |  |  | 0.43 | (-0.01, 0.94) | 0.33 | (-0.02, 0.86) | 0.19 | (-0.01, 0.64) | 0.12 | (0.00, 0.46) | 0.10 | (0.03, 0.16) |
| *High dietary sodium intake* |  |  |  |  |  |  |  |  |  |  |  |  |
| Ischemic heart disease |  |  | 0.09 | (0.06, 0.11) | 0.05 | (0.03, 0.07) | 0.06 | (0.05, 0.08) | 0.03 | (0.01, 0.05) | 0.04 | (0.01, 0.07) |
| Total stroke |  |  | 0.06 | (0.05, 0.08) | 0.06 | (0.05, 0.08) | 0.06 | (0.04, 0.08) | 0.04 | (0.02, 0.06) | 0.05 | (0.03, 0.07) |
| Hypertensive disease |  |  | 0.24 | (0.18, 0.32) | 0.24 | (0.18, 0.32) | 0.27 | (0.20, 0.34) | 0.20 | (0.15, 0.27) | 0.20 | (0.15, 0.25) |
| Other selected CVD |  |  | 0.13 | (0.09, 0.17) | 0.13 | (0.09, 0.17) | 0.14 | (0.10, 0.19) | 0.10 | (0.08, 0.14) | 0.10 | (0.08, 0.13) |
| Stomach cancer |  |  | 0.24 | (-0.02, 0.44) | 0.27 | (0.00, 0.46) | 0.27 | (-0.01, 0.48) | 0.29 | (-0.01, 0.47) | 0.28 | (-0.03, 0.48) |
| *Low fruit and vegetable intake* |  |  |  |  |  |  |  |  |  |  |  |  |
| Ischemic heart disease |  |  | 0.14 | (0.03, 0.23) | 0.08 | (0.02, 0.15) | 0.04 | (0.01, 0.08) | 0.00 | (0.00, 0.00) | 0.00 | (0.00, 0.00) |
| Ischemic stroke |  |  | 0.20 | (0.06, 0.31) | 0.12 | (0.04, 0.20) | 0.07 | (0.02, 0.11) | 0.06 | (0.00, 0.12) | 0.00 | (0.00, 0.00) |
| Colon cancer |  |  | 0.00 | (0.00, 0.00) | 0.00 | (0.00, 0.00) | 0.00 | (0.00, 0.00) | 0.00 | (0.00, 0.00) | 0.00 | (0.00, 0.00) |
| Esophagus cancer |  |  | 0.28 | (0.02, 0.48) | 0.18 | (0.01, 0.32) | 0.10 | (0.00, 0.19) | 0.00 | (0.00, 0.00) | 0.00 | (0.00, 0.00) |
| Lung cancer |  |  | 0.14 | (0.03, 0.23) | 0.08 | (0.02, 0.15) | 0.04 | (0.01, 0.08) | 0.00 | (0.00, 0.00) | 0.00 | (0.00, 0.00) |
| Mouth cancer |  |  | 0.28 | (0.01, 0.48) | 0.18 | (0.02, 0.32) | 0.10 | (0.00, 0.19) | 0.00 | (0.00, 0.00) | 0.00 | (0.00, 0.00) |
| Pharynx cancer |  |  | 0.28 | (0.04, 0.49) | 0.18 | (0.01, 0.32) | 0.10 | (0.01, 0.19) | 0.00 | (0.00, 0.00) | 0.00 | (0.00, 0.00) |
| Stomach cancer |  |  | 0.00 | (0.00, 0.00) | 0.00 | (0.00, 0.00) | 0.00 | (0.00, 0.00) | 0.00 | (0.00, 0.00) | 0.00 | (0.00, 0.00) |
| *Hepatitis B virus* |  |  |  |  |  |  |  |  |  |  |  |  |
| Liver cancer |  |  | 0.28 | (0.19, 0.40) | 0.28 | (0.19, 0.41) | 0.28 | (0.19, 0.40) | 0.30 | (0.19, 0.43) | 0.30 | (0.19, 0.43) |
| *Hepatitis C virus* |  |  |  |  |  |  |  |  |  |  |  |  |
| Liver cancer |  |  | 0.12 | (0.07, 0.20) | 0.36 | (0.25, 0.50) | 0.55 | (0.41, 0.68) | 0.71 | (0.58, 0.81) | 0.73 | (0.61, 0.84) |
| *Helicobacter pylori* |  |  |  |  |  |  |  |  |  |  |  |  |
| Stomach cancer |  |  | 0.31 | (0.19, 0.43) | 0.47 | (0.31, 0.61) | 0.56 | (0.38, 0.68) | 0.58 | (0.42, 0.70) | 0.58 | (0.43, 0.70) |
| *Human papilloma virus* |  |  |  |  |  |  |  |  |  |  |  |  |
| Cervix uteri cancer |  |  | 1.00 |  | 1.00 |  | 1.00 |  | 1.00 |  | 1.00 |  |
| *HTLV-1* |  |  |  |  |  |  |  |  |  |  |  |  |
| Adult T-cell leukemia |  |  | 1.00 |  | 1.00 |  | 1.00 |  | 1.00 |  | 1.00 |  |
| *Joint risk* |  |  |  |  |  |  |  |  |  |  |  |  |
| Ischemic heart disease |  |  | 0.32 | (0.22, 0.56) | 0.59 | (0.46, 0.72) | 0.55 | (0.47, 0.61) | 0.44 | (0.31, 0.54) | 0.40 | (0.27, 0.53) |
| Ischemic stroke |  |  | 0.26 | (0.09, 0.42) | 0.36 | (0.20, 0.51) | 0.28 | (0.18, 0.36) | 0.06 | (0.02, 0.12) | 0.00 | (0.00, 0.00) |
| Total stroke |  |  | 0.22 | (0.12, 0.49) | 0.46 | (0.32, 0.71) | 0.47 | (0.39, 0.55) | 0.28 | (0.17, 0.39) | 0.32 | (0.19, 0.44) |
| Hypertensive disease |  |  | 0.56 | (0.35, 0.82) | 0.94 | (0.79, 0.99) | 0.93 | (0.84, 0.98) | 0.85 | (0.74, 0.94) | 0.81 | (0.72, 0.89) |
| Other selected CVD |  |  | 0.26 | (0.17, 0.37) | 0.63 | (0.47, 0.79) | 0.68 | (0.57, 0.80) | 0.58 | (0.47, 0.70) | 0.55 | (0.48, 0.64) |

CVD, cardiovascular disease; HTLV-1, human T-lymphotropic virus1.

Values in parentheses indicate lower and upper bounds of 95% confidence intervals.
